# *I love my job. But it’s physically*,* mentally*,* and emotionally draining”*: a cross-sectional survey exploring midwives’ intentions of leaving the profession in Melbourne, Australia

**DOI:** 10.1186/s12913-024-11863-7

**Published:** 2024-11-26

**Authors:** Robyn P Matthews, Michelle S Newton, Rebecca L Hyde, Touran Shafiei, Fleur Llewelyn, Della A Forster

**Affiliations:** 1 Judith Lumley Centre, La Trobe University, Bundoora, VIC 3086 Australia; 2 School of Nursing & Midwifery, La Trobe University, George Singer Building, Bundoora, VIC 3086 Australia; 3 The Royal Women’s Hospital, Locked Bag 300, Cnr Grattan St and Flemington Rd, Parkville, VIC 3052 Australia

**Keywords:** Midwives, Attrition, Intention to leave, Burnout, Job satisfaction

## Abstract

**Background:**

Prior to the COVID-19 pandemic there were midwifery workforce deficits reported in Australia, but inadequate workforce data to identify retention and attrition in the profession. In the post-pandemic era, workforce deficits continue. This paper reports on midwives’ intentions to leave the profession and explores reasons for and factors associated with having high intention to leave, to inform strategies that can address retention and attrition of midwives.

**Methods:**

A cross-sectional survey with midwives was conducted in 2017 via an online survey in two maternity care sites in Victoria, Australia. Plans for remaining in or leaving the profession were explored along with reasons for leaving or intending to leave the profession. Other data collected included demographic and workforce characteristics and occupational stressors. Burnout was measured using the Copenhagen Burnout Inventory and job satisfaction using the Midwifery Process Questionnaire. Descriptive statistics, univariate, multivariate analyses, and content analysis were used for data analysis.

**Results:**

Of the 326 respondents (326/508, 64%), over half had considered leaving the midwifery profession in 12 months prior to the study, 20% had thought about leaving frequently and 12% were planning on leaving in the next five years. The main reasons for leaving were not wanting to do shift work, feeling worn out, and experiencing work-related stress. Factors associated with a high intention to leave the *profession* were work-related burnout, poor job satisfaction and a high intention to leave the *workplace*. Age did not impact intention to leave but was influential on the reasons for leaving.

**Conclusions:**

Pre-pandemic, midwives in Victoria, Australia had a high intention to leave the profession regardless of age. Approaches that address midwifery stress, burnout, and fatigue need to be considered, including developing options that offer employment that does not require shift work. To provide safe quality care that supports positive outcomes for women and their families, an appropriate midwifery workforce must be achieved and maintained. Understanding midwives’ intentions to leave the profession is critical and requires ongoing attention given the workforce is likely to remain under significant stress until the major contributing factors are addressed.

**Supplementary Information:**

The online version contains supplementary material available at 10.1186/s12913-024-11863-7.

## Introduction

Addressing attrition from the midwifery profession is of vital importance to maintaining robust and sustainable maternity care in Australia and around the globe. Quality care facilitates optimal outcomes for women and their infants but is dependent on an adequately skilled and staffed midwifery workforce [1]. There is increasing evidence over the past two decades that the midwifery profession has a retention issue leading to workforce shortages globally [2]. The 2021 annual international report, the *State of the Midwifery Workforce* details a global shortage of 900,000 midwives [2]. In Australia there is no available accurate governmental reported data on midwifery workforce shortages [3], but there is documented evidence of ongoing deficits prior to the COVID-19 pandemic [4, 5] that worsened during and after the pandemic [6]. While the pandemic is likely to have added considerable stress to the midwifery workforce [2, 7], understanding what impacted retention and attrition of midwives pre-pandemic can provide an important baseline from which to start to develop strategies to increase retention of midwives.

In Australia midwifery has been considered an ageing profession – national data shows the average age of a midwife in 2017 was 48 years [8], and a study published in 2021 predicted a significant decline in the number of midwives in Australia between 2018 and 2023 as many midwives approach retirement [3]. Australian midwifery workforce data is relatively inaccurate due to low responses to workforce surveys and challenges in differentiating between midwives who are currently working in midwifery roles and those who have a midwifery registration but are no longer working as midwives [3]. There are no consistently reported state or national workforce data that predict future workforce needs or identify where (or if) there are retention and attrition issues in the midwifery workforce [3]. There has been a 33% increase in the number of midwifery students graduating since 2012 in Australia [9], which is theoretically adequate to replace retiring midwives [10], but midwifery workforce deficits persist [4, 5]. The reasons for this are not clear, but likely to be related to workforce attrition due to reasons other than retirement.

We identified five studies that explored midwives’ intentions, reasons for and factors associated with considering leaving the midwifery profession prior to the COVID-19 pandemic (Table S1) [11–15]. Two were conducted in Australia [11, 12], one each in the United Kingdom (UK) [14], Canada [13] and the Netherlands [15]. Most of the studies used a cross-sectional design [11–15] (one included data from two different groups: interviews with former midwives and a cross-sectional survey for midwives who were currently working to explore their intention to leave [15]), with low response rates (14% [13], 16% [14], 21% [11]) or response rates not stated [12, 15]. Most of the studies included predominantly older or more experienced midwives [11, 12, 14]. Midwives’ intention to leave the profession (the percentage who had thought about leaving the profession) varied from 34 to 67% [11–15], with between 24% and 29% planning to leave in the next five years.

The two Australian studies reported 43% [11] and 46% [12] of midwives had thought about leaving the midwifery profession. Across all studies the most commonly reported reasons for intending to leave were family commitments [11, 12, 15], career change [11, 12], dissatisfaction with the organisation of midwifery care [12, 14, 15], and ill health or concerns about mental/physical health [12, 15] (Table 1). There were mixed findings related to factors associated with intention to leave in the two Australian studies; one study found that older and more experienced midwives were most likely to be intending to leave the profession [11], and the other found that midwives aged under 40 were most likely to leave [12]. For midwives under 40 years of age, the main reasons for wanting to leave were dissatisfaction with the organisation of midwifery or dissatisfaction with their current midwifery role [12].

There are limitations with the above studies – while some had large sample sizes, they represented a small percentage of the population of potential respondents (this information only provided in three studies [11, 13, 14]), so there is potential for sample bias (e.g., those most likely to be planning to leave might be more or less likely to participate in the survey) which may affect the generalisability of the findings. The midwifery profession is also practiced very differently in Canada and the Netherlands compared with Australia and the UK. In Canada and the Netherlands midwives practice mostly in a self-employed capacity in on-call models [13, 15], and the Netherlands study included community midwives only [15]. In Australia and the UK, midwives are more likely to work in fragmented predominantly hospital-based rotating shift work models [16, 17]. Some of the reasons for intending to leave may therefore not be generalisable across different countries. The study recruitment methods (e.g., via professional organisations [12–14]), along with the location and types of work participants were engaged in may also have led to selection bias, which potentially influences generalisability in these studies.

In Australia, the midwifery workforce deficits are in a context of an ageing workforce, but with a continued increase in the number of graduating midwifery students [9]. This indicates that there may be increasing attrition unrelated to retirement. If workforce retirement predictions are accurate and there is a significant decline in the number of more senior midwives, there may not be skilled and experienced midwives to replace them. Identifying what issues exist, understanding current midwives’ intentions to stay in or leave the profession, and exploring what factors affect this (outside of retirement) is critical. The included studies provide little evidence as to why midwives who are not at retirement age are considering leaving the profession. Most of the studies included midwives who had *any* intention to leave the profession (had thought about leaving ‘at all’), however a focus on midwives who have a *high* intention to leave (i.e., think about it regularly) may allow for a better understanding of who is at most risk of leaving the profession.

The aims of this study are:

1. Identify the prevalence of midwives intending to leave the profession in a population of midwives working in two maternity services in Melbourne, Victoria.
2. Explore associations between age and intention to leave the profession.
3. Explore factors associated with a *high* intention to leave the profession.
4. Explore reasons behind intention to leave the profession.
5. Use findings to inform strategies to address retention and attrition of midwives in the midwifery profession.

## Methods

### Design

This paper is the third in a series exploring midwives’ experiences of work, from a larger study called ‘EXPert – Exploring midwives’ and nurses’ perceptions of ‘expertise’ and experiences of work’ [18, 19]. The ‘EXPert study’ used a cross-sectional design administered via an online survey (which was created specifically for this study) to explore the concept of an ‘expert’ midwife or nurse, and to gain an understanding of midwives’ and nurses’ views and experiences of work and their work context (survey in Supplementary File 3).

### Setting

The study was conducted at two sites of a maternity service in Melbourne, Victoria. Site one is a maternity service in a large tertiary women’s hospital that provides maternity care for over 7,700 women with low and high-risk pregnancies and is a referral service for other hospitals in the state of Victoria. Site one employed 266 permanent midwives at the time of the study. Site two is a maternity service co-located within a general hospital in the outer suburbs of Melbourne with an annual birth rate of 1,400 babies and providing care for women with predominantly low-risk pregnancies and babies born at 34 weeks gestation or above. Site two employed 69 permanent midwives at the time of the study. Both sites were managed by the same organisation. The organisation employed 173 midwives casually across both sites. Most midwives at both sites work roster-based shift work, rotating between areas of maternity care. Site one had a small number of midwives working in an on-call caseload midwifery model. Site one is characteristic of any major maternity service in a capital city in Australia, site two is similar to many small maternity services in metropolitan areas and large regional areas. We have no way of knowing if the workforce culture and workload at the two sites would be generalisable to other maternity services across Australia, but it is likely findings may be less generalisable to small rural or remote maternity services.

### Participants and recruitment

All midwives and nurses permanently or casually employed at either or both sites (*n* = 508) at the time of the study, and working in clinical or non-clinical roles, were eligible to participate. Any midwife or nurse who contributed to the study design or was an investigator on the study was ineligible (*n* = 7). Three of the investigators in this study were also employed clinical midwives in site one and one investigator was employed as part of the clinical education team at site one. The rest of the investigator team were not employed at either site. This paper includes data from midwives who were working in either or both services at the time of the study.

An invitation to participate in the study were sent via an email from the Executive Director of Nursing and Midwifery, with a public link to an online survey and an information statement describing the study. As the survey was anonymous (survey responses were not linked to email addresses or any identifying information), all participants were sent four reminder emails at two-week intervals. Strategies used to encourage participation included posters, and research team members promoting the study at clinical handovers, in-service education, and team meetings at both sites.

### Data collection

The study questionnaire was administered using an online survey. Prior to the development of the data collection tool, seven focus groups were conducted, and included a total of 53 midwives and nurses (including participants in clinical, non-clinical and management roles). The themes from the focus group discussions were used in the development of the survey questions.

Demographic and work history data (e.g., age, caring responsibilities, years qualified as midwife, type of work hours, areas worked in the maternity unit etc.) were collected using specifically designed questions, and other questions developed based on the literature and themes from the focus groups.

#### Measures of occupational stressors

A number of potential occupational stressors were identified that could impact intention to leave the profession. The stressors included potential for fatigue at work, poor perception of skill mix, burnout, lack of job satisfaction, lack of acknowledgement, lack of support to fulfil the midwifery role, and poor relationships with senior staff. To explore midwives’ views and experiences of the identified occupational stressors, the survey included a series of specifically designed questions as well as existing validated tools (See Supplementary 3 (S3) for survey).

- *Potential for fatigue* at work was measured using a specifically developed question, *“Currently*,* in an average week at this maternity service how often would you get your scheduled breaks on each shift/day of work?’* Responses were ‘100% of shifts’, ‘75% of shifts’, ‘50% of shifts’, ‘25% of shifts’, ‘10% of shifts’ and ‘0% of shifts’ (question 3.27 S3).
- *Perception of skill mix* was measured using a specifically developed question: *“Currently*,* in an average week at this maternity service in your opinion how often is the staff skill mix on each shift ‘unsafe’?”* Responses were ‘None of the time’, ‘Little of the time’, ‘Some of the time’, ‘Most of the time’, and ‘All of the time’ (question 3.07 S3).
- *Burnout* was measured using the Copenhagen Burnout Inventory [20] – a 19-item validated tool that identifies burnout in relation to three domains: ‘Personal’ burnout (six items), ‘Work-related’ burnout (seven items), and ‘Client-related’ burnout (six items) (question 3.26 S3). It uses a five-point Likert scale for 10 of the items (Never = 0, Seldom = 25, Sometimes = 50, Often = 75, Always = 100) and for the remaining nine items (To a very low degree = 0, To a low degree = 25, Somewhat = 50, To a high degree = 75, and To a very high degree = 100). Scores are calculated by the mean of total items for each domain. A score of 50 or above signifies burnout in that domain [20]. For a more detailed discussion of this scale use in the ‘EXPert study’ please refer to previous work [18].
- *Job satisfaction* was measured using the Midwifery Process Questionnaire [21] – a 20-item validated tool that measures midwives’ views of their work in four domains – ‘Professional satisfaction’ (six items), ‘Professional support’ (five items), ‘Client interaction’ (five items), and ‘Professional development’ (four items) (question 3.32 S3). Half the items were positively worded to reduce a response bias. It uses a five-point Likert scale to measure each item (Strongly disagree=-2, Disagree=-1, Not sure = 0, Agree = 1 and Strongly agree = 2). Scores were added together and averaged for each of the domains (after negatively worded statements were reverse scored). Scores ranged from − 2 to 2, under 0 was considered a negative attitude towards that domain, 0 a neutral attitude and over 0 a positive attitude towards that domain. For a more detailed discussion of this scale use in the ‘EXPert study’ please refer to previous work [19].
- *Acknowledgement at work *was measured using a specifically developed question: “Do you feel that you are adequately acknowledged by this organisation for the work that you do?” Responses were ‘Yes’ or ‘No’ (question 3.37 S3).
- *Support at work *was measured using a specifically developed question: “Do you feel that you need more support in order to fulfil your current role?” Responses were ‘Yes’ or‘No’ (question 3.12 S3).
- *Relationships with senior staff *were measured using the following specifically developed statement: “I am worried about approaching senior staff for assistance”. Responses were measured using a five-point Likert scale (‘Never’, ‘Rarely’, ‘Sometimes’, ‘Often’, ‘Almost always’) (question 3.09 S3).

#### Measures of ‘Intention to leave the ‘workplace’ and ‘profession’ and ‘reasons why’

Intention to leave the *workplace* was identified in the literature as potentially impacting intention to leave the *profession*.

- Intention to leave the *workplace *was measured by the following specifically designed question “In the last 12 months how often have you thought about leaving this maternity service?” Responses were ‘Never’, ‘Occasionally (a couple of times per year)’, ‘Sometimes (monthly)’, ‘Frequently (weekly)’ and ‘All the time’ (question 1.42 S3).
- Intention to leave the *profession *was measured by the following specifically designed questions: “How long do you plan to continue working in the profession of midwifery?” with responses‘<1 year’, ‘1-2 years’, ‘3-5 years’, ‘6-10 years’, ’11-20 years’, ‘>20 years’ and ‘Not sure’ (question 1.44 S3)  and “In the last 12 months have you considered leaving the profession of midwifery?” Responses were ‘Never’, ‘Occasionally (a couple of times per year)’, ‘Sometimes (monthly)’, ‘Frequently (weekly)’ and‘All the time’ (question 1.45 S3).
- Reasons for considering leaving the *profession *was measured with a list of 13-items of potential reasons. Only midwives who provided any response other than ‘Never’ to the intention to leave the profession question answered this. Midwives could tick all items that applied and there was an ‘Other’ option to capture any other reasons not listed (question 1.46 S3). The midwives were also given an option to complete an open-ended question with a free-text response which stated “Please feel free to comment further – this is a really important issue and we are very keen to have your thoughts”.

The survey was piloted with midwives external to the study sites and amended as required.

### Data management and analysis

Once finalised, the questionnaire was uploaded to the Research Electronic Data Capture (REDCap) [22] platform, then tested for functionality.

Quantitative data were downloaded and imported into STATA Version 17 [23]. Data cleaning included checks for missing data, expected range, and logic checks. Any inconsistencies in the data were assessed by two members of the research team (RM, DF) and decisions agreed upon by both.

Descriptive analyses were undertaken to describe the characteristics of the participants and report on intention to leave the profession and reasons why midwives were considering leaving the profession. Sub-analyses were undertaken by grouping midwives into age categories (‘≤30 years’, ’31–45 years’, ’46–54 years’, ‘≥55 years’) and using those categories to further explore intention to leave the profession and reasons why midwives were considering leaving the profession. Any midwife who did not provide a response to the question on age was not included in these sub-analyses.

Each of the occupational stressors (excluding validated scales) were organised into dichotomised variables (if not already a ‘Yes/No’ option) for use in further analysis and to make it easier to understand and interpret the findings (Table 1). The ‘intention to leave’ variable was then dichotomised into ‘Never or occasionally’ to indicate a low intention to leave the profession and ‘Sometimes, frequently and all the time’ to indicate a high intention to leave the profession for inclusion in the logistic regression analyses.

The Copenhagen Burnout Inventory and the Midwifery Process Questionnaire were both coded as per the authors instructions [18, 19].

**Table 1:** Variables dichotomised for analysis

Variable	Aggregated/dichotomised variables/responses	
**Able to take regular breaks** *“Currently, in an average week at this maternity service how often would you get your scheduled breaks on each shift/day of work?”*	**Yes** (‘100% of shifts’, ‘75% of shifts’)	**No** (‘50% of shifts’, ‘25% of shifts’, ‘10% of shifts’ and ‘0% of shifts’)
**Perception skill mix is unsafe** *“Currently; in an average week at this maternity service in your opinion how often is the staff skill mix on each shift ‘unsafe’?”*	**Yes** (‘Some of the time’, ‘Most of the time’, and ‘All of the time’)	**No** (‘None of the time’, ‘Little of the time’)
**Worried about approaching senior staff** *“I am worried about approaching senior staff for assistance”*	**Yes** (‘Sometimes’, ‘Often’, ‘Almost always’)	**No** (‘Never, ‘Rarely’)
**Intention to leave the workplace** *“In the last 12 months how often have you thought about leaving this maternity service?”*	**High intention to leave** (‘Sometimes (monthly)’, ‘Frequently (weekly)’ and ‘All the time’)	**Low intention to leave** (‘Never’, ‘Occasionally (a couple of times per year)’)
**Intention to leave the profession** *“In the last 12 months have you considered leaving the profession of midwifery?”*	**High intention to leave** (‘Sometimes (monthly)’, ‘Frequently (weekly)’ and ‘All the time’)	**Low intention to leave** (‘Never’, ‘Occasionally (a couple of times per year)’)

Univariate regression analyses were conducted to explore associations between the *high* intention to leave the profession variable and participants’ demographic and work characteristics (age, children, years post qualification, work area) and occupational stressors (potential for fatigue, perception of skill mix, burnout, job satisfaction, acknowledgement at work, support at work and relationships with senior staff) in their dichotomised format. Results are presented as odds ratios (OR) and 95% confidence intervals (CI).

To conduct multivariable regression analyses, we used any variables found in the univariate analyses with a Wald statistic p-value of ≤ 0.2 to create a multivariable model. At each stage, the variable that had the lowest association with the outcome variable in the model was eliminated. This was done one variable at a time, creating new models in successive regressions. The likelihood ratio test was used with each succeeding model to check it did not differ substantially from the previous model except for the removal of the previous variable. New models were created until only variables with a Wald statistic of p-value of ≤ 0.05 remained. Results are presented as adjusted odds ratio (adj OR) and 95% confidence intervals (CI). Secondary multivariable regression analysis was conducted including only site one respondents to try and understand if there were differences based on workplace setting. We did not conduct a secondary multivariable regression analysis including only site two respondents as the sample size for that group was too small.

Free-text responses from the open-ended questions were downloaded into a Microsoft Excel file. Inductive content analysis [24] (an approach that includes open coding, creating categories and abstracting themes [25]) was conducted by two members of the research team (RM, MN). Each researcher independently grouped comments into codes, categories, and then into themes, then discussed the codes and themes together and identified any similarities or differences. Once agreement was reached by the initial researchers coding the data, the global theme and sub-themes were presented to the entire research team to check on agreement. Trustworthiness was ensured by using two independent researchers to initially conduct content analysis individually, then meeting to ensure agreement and further assessing credibility by ensure agreement with the themes by the entire research team. The codes and themes were also re-analysed by the age group of participants to identify any patterns. Themes are presented descriptively and supported by illustrative quotes.

### Ethical considerations

Ethical approval was sought through the participating study site hospital and university, but it was judged that this project met the National Health and Medical Research Council requirement for being a quality assurance/audit project (in accordance with the Australian Government National Statement on Ethical Conduct in Human Research 2007 [26]). As such it was approved by the University Ethics Committee (approved 4th April 2016) and the study sites Research Committee and Human Research Ethics Committee (approved 3rd May 2016). All potential respondents of the participating maternity services were informed about the goal, procedures, risks, benefits, anonymity of data and alternatives for participating in the study via written study information included in the email invitation, via discussion in clinical handovers, in-service education, and team meetings, and via paper flyers so that they could make an informed decision about participation. Participation in the survey was considered as having obtained informed consent from participants.

## Results

The survey opened 16th January 2017 and closed 12th March 2017. The survey invitation was distributed to a total of 508 midwives (both permanently and casually employed) across both sites. The overall response rate was 64% (326/508). Of the permanently employed midwives, the response rate was 95% (252/266) from site one and 38% (26/69) from site two. Of the casually employed midwives from both sites the response rate was 28% (48/173) (some casual midwives worked across both sites therefore this group can only be described collectively).

### Demographics and workforce characteristics

The largest proportion of the midwives were aged ≤ 30 years (42%), and 71% were aged 45 years or younger (Table 2). Participants’ median age was 33 years (range 23–72 years). A third were single, 44% had children and 7% were carers for someone other than children. Almost half (46%) of the midwives were ≤ 5 years post qualification, with a median of six years and a mean of 10 years.

**Table 2:** Demographic and work characteristics of all respondents

Characteristic		*n*	%
**Age (*n*** = **317)**			
≤ 30 years		133	*42*
31–45 years		92	*29*
46–54 years		46	*15*
≥ 55 years		46	*15*
Median (years) *range*		33	*23–72*
Average (mean) age (SD)		38	*± 13*
**Relationship status (** ***n*** **= 311)**			
Single		100	*32*
De facto/Married		211	*68*
**Have children (** ***n*** **= 322)**		143	*44*
**Carer for someone other than children (** ***n*** **= 326)**			
No		304	*93*
**Carer for someone other than children (** ***n*** **= 326)**		22	*7*
**Years post qualification (** ***n*** **= 316)**			
≤ 5 years		147	*46*
6–10 years		63	*20*
> 10 years		106	*34*
Median (years) *range*		6	*1–38*
Mean (years) *SD*		10	*10*
**Work site (** ***n*** **= 326)**			
Site one		298	*91*
Site two		26	*8*
Both		2	*0.6*
**Type of work hours (** ***n*** **= 326)**			
Non shift work (Monday-Friday/permanent nights/on-call)		66	*20*
Shift worker		260	*80*
**Work area (** ***n*** **= 322)**			
Rotate to all/most areas (e.g., postnatal, birth centre, antenatal clinic)		279	*87*
Works in one area only (no rotations)		43	*13*
**Currently working elsewhere (in addition to study sites) (** ***n*** **= 325)**		56	*17*

As described in the methods the two sites differed in terms of patient population and level of care provided, so the demographic and work characteristics were reviewed by site to see if there were any differences. A higher percentage of midwives from site two were ≥ 35 years (77% vs. 44%), had children (71% vs. 42%) and were more than ten years post qualification (54% vs. 32%). There were no other differences in characteristics between midwives from the two sites.

### Occupational stressors

Almost two-thirds (65%) of midwives were experiencing personal burnout, 47% work-related burnout and 8% client-related burnout at the time of the study (Table 3). Fifteen percent of midwives scored negatively towards their professional satisfaction (how they felt about being a midwife) and 51% scored as having a negative attitude towards their professional support (the amount of time they can spend with women, the stress they experience and support from other colleagues). Almost half (47%) the midwives had a negative attitude score on their interaction with clients (their ability to provide women with choice, continuity, and individualised care) and 21% scored negatively towards their professional development (opportunities to develop skills and further their professional education).

Almost one third (31%) of midwives reported *not* being able to take regular breaks in an average week and two-thirds of midwives felt the skill mix in an average shift to be unsafe (Table 4). Most midwives (63%) did not feel adequately acknowledged by the organisation for the work they do and almost half (42%) wanted more support to be able to fulfil their current role. Over a quarter (27%) of midwives felt worried about approaching senior staff for assistance.

**Table 3:** Occupational stressors

Stressor		*n*	%
**Burnout (burnout score ≥ 50)**			
Personal burnout (*n* = 241)		156	*65*
Work-related burnout (*n* = 240)		113	*47*
Client-related burnout (*n* = 237)		20	*8*
**Midwifery Process Questionnaire (negative attitude score < 0)**			
Negative attitude towards professional satisfaction (*n* = 215)		32	*15*
Negative attitude towards professional support (*n* = 222)		114	*51*
Negative attitude towards client interaction (*n* = 214)		101	*47*
Negative attitude towards professional development (*n* = 224)		46	*21*
**Not able to take regular breaks (** *n* **= 236)**		73	*31*
**Consider the skill mix to be unsafe (** *n* **= 249)**		167	*67*
**Do not have adequate acknowledgment from the hospital (** *n* **= 228)**		144	*63*
**Need more support to fulfil current role (** *n* **= 244)**		102	*42*
**Worried about approaching senior staff (** *n* **= 241)**		64	*27*

### Intention to leave the workplace and/or profession

**Fig. 1:**
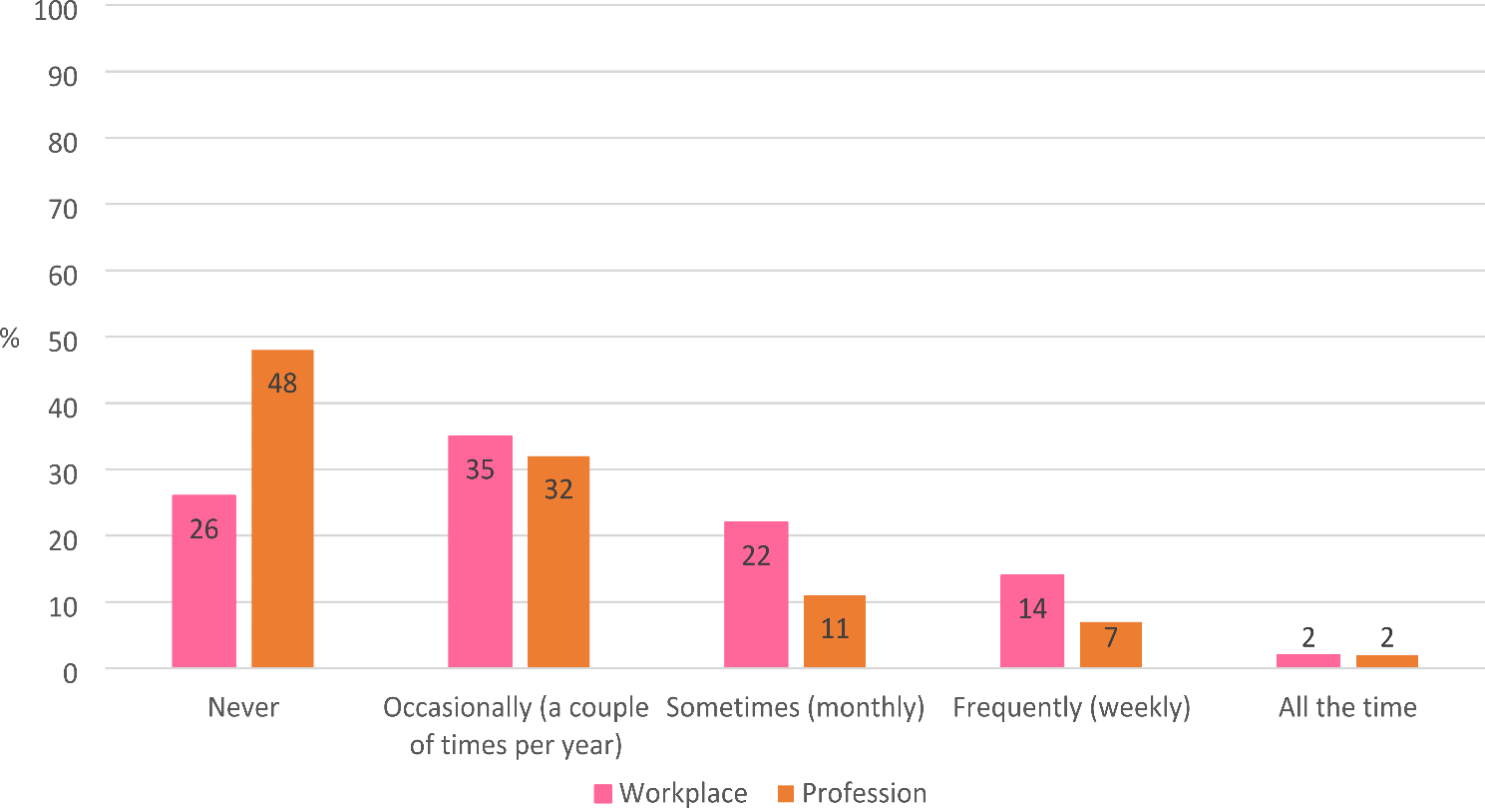
How often had considered leaving the workplace (*n* = 322) and the profession (*n* = 323) in 12 months prior to survey

Midwives were asked how often they had thought about leaving their *workplace* in the 12 months prior to the survey. Almost 40% reported a *high* intention to leave their *workplace* (i.e., had thought about it monthly, weekly or ‘all the time’) (Fig. 1). More than half (52%) the midwives had considered leaving the *profession* in the 12 months preceding the survey. One in five midwives (20%) had a *high* intention to leave the *profession*.

**Fig. 2:**
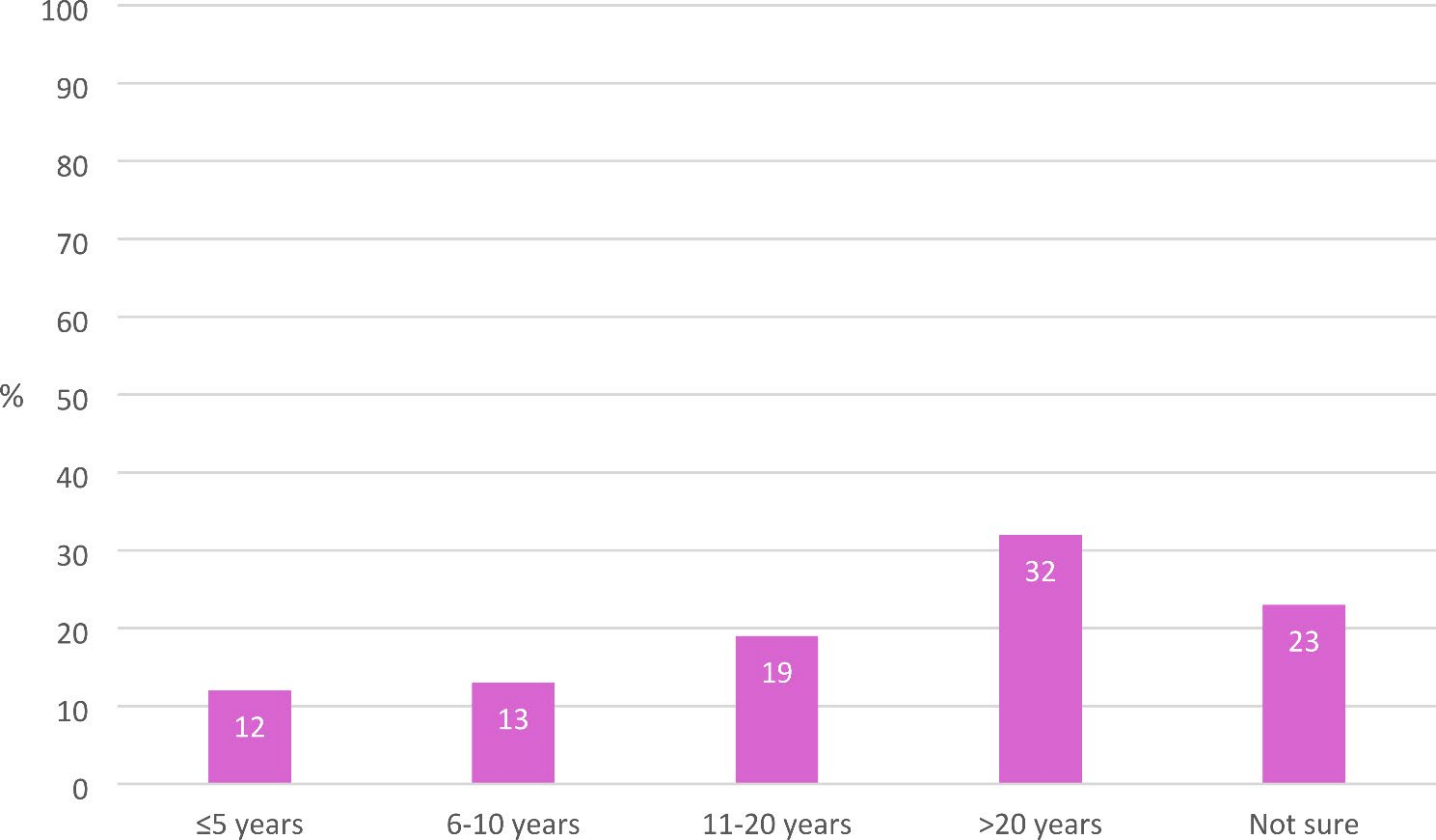
How long planned to continue working in midwifery (*n* = 324)

Twelve percent of the midwives (*n* = 40) were planning on leaving the profession in the next five years and nearly a quarter (23%) were unsure how long they would stay in the profession (Fig. 2).

Midwives that reported they had thought about leaving the profession were given a list of potential reasons why and asked to tick all that applied, as well as having the option of ‘Other, please describe’. Of those midwives, 75% (127/169) provided one or more reasons as to why they were considering leaving the profession (Table 4). The most common reasons were ‘not wanting to work shift work’ (63%), feeling ‘worn out’ (57%), ‘work related stress’ (54%), ‘disillusionment with midwifery’ (47%), and ‘wanting to work set days’ (40%) (Table 4). Fourteen (11%) midwives reported retirement as a reason they intended to leave the profession.

**Table 4:** Reasons for wanting to leave the profession

Reasons	*n* (*n* = 127)	%
Not wanting to work shift work	80	*63*
Worn out	72	*57*
Work related stress	69	*54*
Disillusionment with midwifery	60	*47*
Wanting to work set days	51	*40*
Earn more money in other professions	38	*30*
Want a career beyond nursing and midwifery	23	*18*
Not conducive to raising a family	22	*17*
‘Nothing left to give’	17	*13*
Health concerns (e.g. injury preventing continuing working)	15	*12*
Retirement	14	*11*
Mental health issues	10	*8*
No longer challenged	2	*2*
Other (occupational violence, rosters, lack of acknowledgement)	3	*2*

**Fig. 3:**
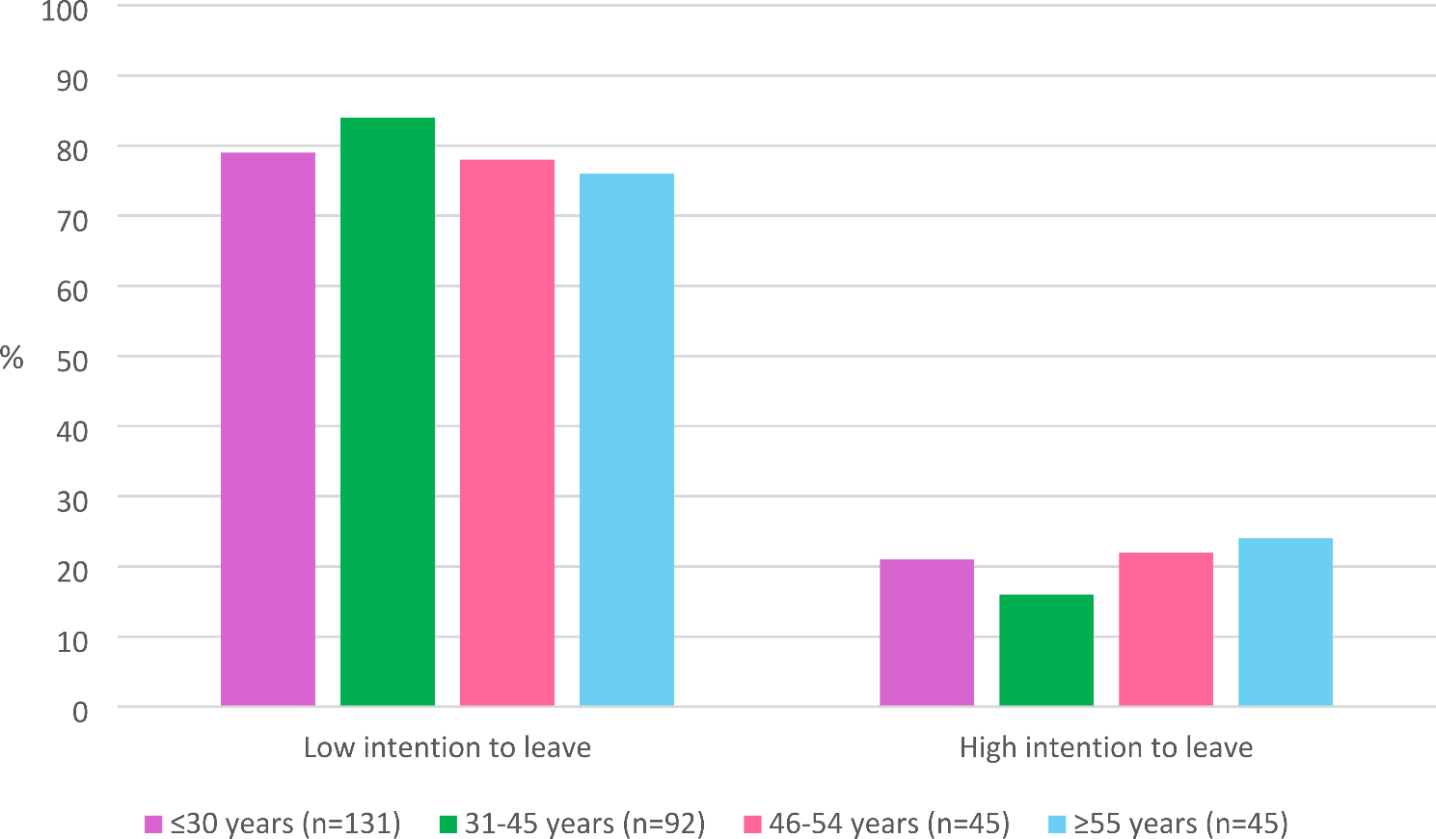
Intention to leave the profession by age group, dichotomised as low and high intention

Figure 3 shows the proportion of midwives with low and high intention to leave the profession by age group.

**Fig. 4:**
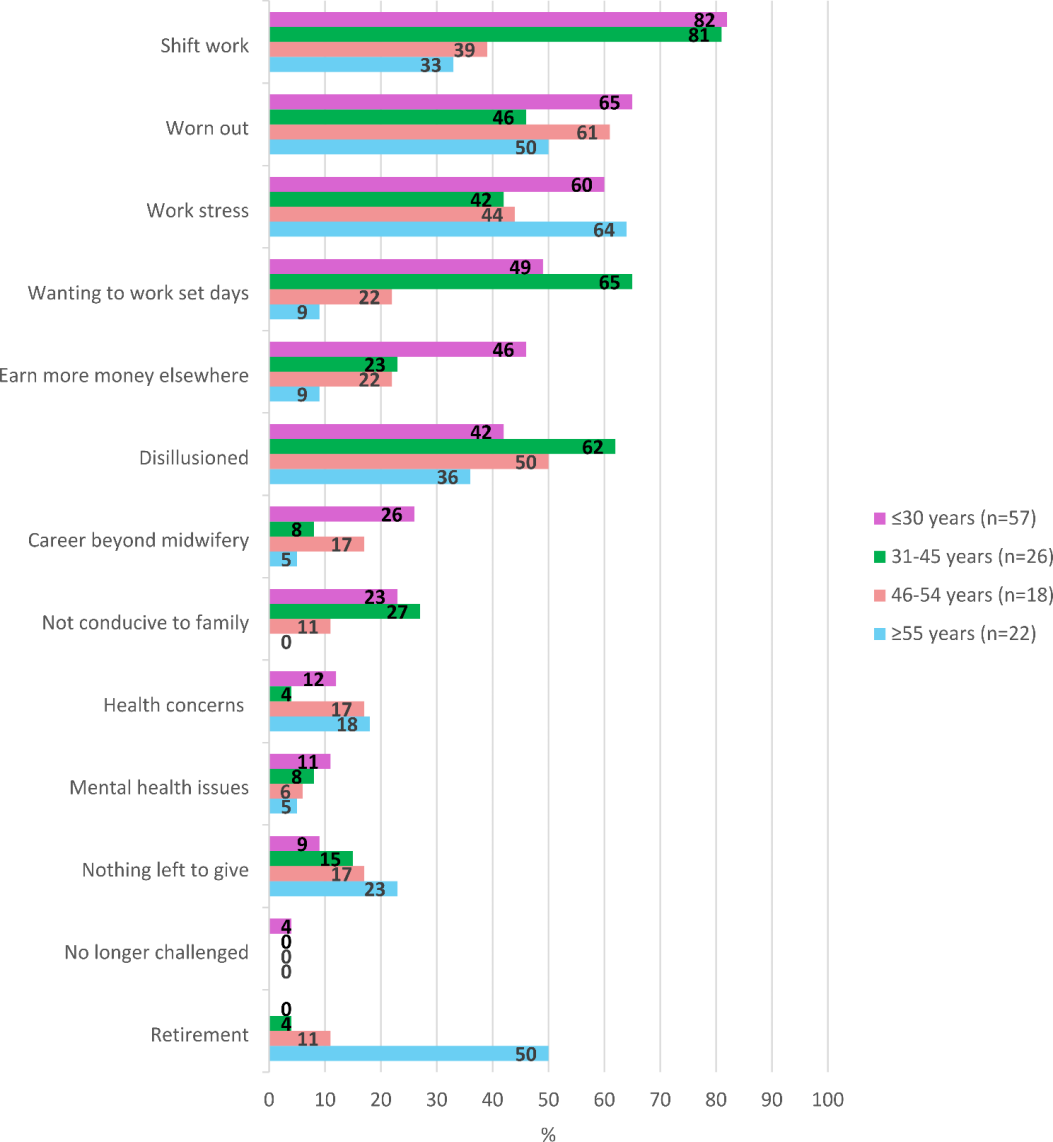
Reason for leaving the profession, by age group (%)

The main reasons for considering leaving the profession varied by age (Fig. 4). For midwives aged ≤ 45 years, ‘not wanting to work shift work’ was the most common reason, for those aged 46–54 years it was feeling ‘worn out’ (61%), and for those aged ≥ 55 years, ‘work-related stress’ was the main reason for considering leaving midwifery (64%). Nearly half of those aged ≤ 30 years felt they could ‘earn more money elsewhere’ but it was not a highly ranked issue for the other three groups.

### Factors related to/associated with intention to leave the profession

We conducted univariate analyses of the ‘intention to leave’ the *profession* variable with the demographic characteristics, workforce characteristics and occupational stressors. Midwives that reported ‘retirement’ (*n* = 14) as a reason for planning to leave the profession were removed from the univariate and multivariable analyses because we considered it to be a normal part of career progression.

In the univariate analysis, no demographic or work characteristics were associated with *high* intention to leave the *profession* (Table S2). The occupational stressors that were associated with a *high* intention to leave the *profession* were: experiencing personal burnout (OR 6.50, 95% CI 2.23, 18.98), work-related burnout (OR 6.43, 95% CI 2.82, 14.67), client-related burnout (OR 5.41, 95% CI 2.08, 14.04), having a negative attitude to professional satisfaction (OR 7.65, 95% CI 3.32, 17.63), a negative attitude towards their professional support (OR 3.07, 95% CI 1.44, 6.52), a negative attitude towards their professional development (OR 2.19, 95% CI 1.04, 4.62), having inadequate acknowledgement from the organisation (OR 4.24, 95% CI 1.70, 10.58) and having a *high* intention to leave the *workplace* (OR 14.54, 95% CI 6.78, 31.18).

A multivariable analysis was then conducted. Any variable in the univariate analysis with a Wald statistic p-value ≤ 0.2 was included in the analysis. The initial multivariable model included experiencing personal burnout, work-related burnout, client-related burnout, having a negative attitude towards their personal satisfaction, professional support, client interaction, and professional development, *not able* to take regular breaks, having inadequate acknowledgement from the organisation, needing more support to fulfil their current role, and having a *high* intention to leave the *workplace*. Only midwives that responded to all those variables and the intention to leave the *profession* variable were included in the model (*n* = 176). In the final model the variables that remained associated with a *high* intention to leave the *profession* were: experiencing work-related burnout (adj. OR 4.03, 95% CI 1.20, 13.53), having a negative attitude towards their professional satisfaction (adj. OR 3.08, 95% CI 1.09, 8.68) and having a *high* intention to leave their *workplace* (adj. OR 13.92, 95% CI 3.83, 50.52) (Supplementary File: Table S2).

As the midwifery characteristics differed between the two sites, the univariate and multivariable analysis were re-analysed excluding the midwives from site two (the smaller group) to see if removing that site from the analysis changed the factors impacting intention to leave the profession. In this secondary analysis the same factors reported above remained associated in both the univariate and multivariable analysis. As site two had only a small population, regression analysis was not conducted on that population alone.

#### Responses to open-ended question

Those who indicated that they had thought of leaving the *profession* were given an option to comment further on their reasons why. Sixty-eight participants provided further free-text responses. Their ages ranged from 23 to 65 years, with almost half (32/68) aged ≤ 30 years. There were 62 respondents from site one, and 53 were employed permanently. The global theme identified from the analysis was *‘I love being a midwife*,* but…’*, from which six sub-themes were: *‘Impact of shift work and night duty on health*,* wellbeing and work/life balance’*, ‘*The physical and emotional toll of the work’*, *‘Increased workload impacting on care’*, *‘Care lacking quality leading to job dissatisfaction*’, *‘Experiencing a negative workplace culture*’ and *‘Remuneration not matching effort*’. Overall, midwives’ reasons for leaving the profession were multifaceted, and their comments often reflected a number of the sub-themes.

#### 'I love being a midwife, but….'

Many midwives spoke of their love of being a midwife or working in an environment with a positive atmosphere. They spoke of the privilege of being a midwife and feeling like they made a difference for women and their families.

*“At times it can feel that I give a lot myself*,* my time*,* care and energy and love. This I do by choice*,* I do it because I love the profession and feel like it is such a privileged position to be sharing these tender and vulnerable moments with women*,* new babies*,* and their families.”* (ID 498, aged 30, 4 years’ experience, casual).

However, comments relating to this joy and love of the profession, or the organisation were mostly followed by a ‘but’. They loved their work, *but* they were concerned how sustainable this profession was for them and expressed sadness and frustration in considering leaving the profession. Several sub-themes reflected concern that the respondents had over the feasibility of remaining in the midwifery profession long-term.

#### 'Impact of shift work and night duty on health, wellbeing and work/life balance’

The first sub-theme related to the impact of shift work and night duty on midwives’ overall health, wellbeing, and ability to maintain a work/life balance. Twenty-four midwives made comments that contributed to this sub-theme. For some, working night duty left a heavy toll on them, the swapping back and forth from nights to days frequently caused exhaustion and affected their wellbeing.

*“Although I love my job and am so grateful for the experience that comes with working in an exceptional hospital… I am not coping well with the frequency of night shifts. I find they strongly affect my mental and physical health.”* (ID 378, aged 27, 2 years’ experience, permanent).

For others, shift work in general was an issue which included lack of flexibility with rosters, no option of working set shifts, rostering that impacted their social life, and feeling that the hours they worked were not sustainable for their families. All these factors contributed to thoughts of leaving the profession.

*“Difficulty juggling raising a young family*,* with no outside support and hours of childcare/school. The feeling that you are being difficult needing set days/shifts.”* (ID 536, aged 39, 7 years’ experience, casual).

#### 'The physical and emotional toll of the work’

The second sub-theme was about the difficulty of remaining in the profession due to the physical and emotional toll the work itself had on midwives (22 commented on this). Many midwives spoke of their work causing burnout and exhaustion, being affected both physically and mentally by the work, experiencing work-related stress and feeling anxiety about the responsibility that was associated with being a midwife.

*“I feel as though the physical demands of the job combined with the busyness of the hospital and lack of meal breaks contributes to burn out of midwives. I commit every working day to give everything I have emotionally and physically to the women and babies I care for and I feel as though it is often to my own detriment.”* (ID 500, aged 25, 4 years’ experience, permanent).

*“I love my job. But it’s physically*,* mentally*,* and emotionally draining”* (ID 424, 56 years old, 25 years’ experience, permanent).

#### 'Increased workload impacting on care’

The third sub-theme related to concern that the increased workload midwives were experiencing was impacting on care (22 comments related to this). Contributing factors included the increased acuity of women, there was not being enough staff to care for women and babies, too much administrative work, working above ratios (the state-government legislated minimum number of midwives to number of patients on a ward), and the staff mix being disproportionately junior which could lead to midwives’ feeling unsafe while providing care.

*“Not being able to give appropriate care to women due to poor skill mix*,* repeatedly understaffed and increasingly complex patients while working outside of patient-staff ratios.”* (ID 225, 32 years old, 5 years’ experience, casual).

*“The work loads are getting busier*,* and our main focus is the paperwork and not the women.”* (ID 485, aged 39, 17 years’ experience, permanent).

#### 'Care lacking quality leading to job dissatisfaction’

Comments relating to lack of quality care leading to job dissatisfaction were made by 23 respondents. The inability to provide quality care or provide care in a way that was consistent with the midwives’ underlying philosophy affected their job satisfaction and led to feelings of disillusionment with midwifery. Midwives cited the lack of time to spend with women, the lack of autonomy, and a lack of woman-centred care, and some believed they could not make a difference on a larger scale. Midwives also felt that organisations were focusing on cost rather than quality of care, that there was a lack of necessary resources, and that the overmedicalisation of maternity care was contributing to a cascade of unnecessary interventions.

*“Being at births and feeling disenchanted with what we are doing to women when the mentality we drill into them ‘think about the baby’s well-being’ etc women become self-sacrificing in a way that we forget that woman is a human being too not a vessel we can do as we please”* (ID 358, aged 28, 7 years’ experience, casual).

*“The care for patients seems to be getting worse. The main focus seems to be all about the hospital budget*,* and we are forgetting about the patient and their family.”* (ID 529, aged 45, 17 years’ experience, permanent).

#### 'Experiencing a negative workplace culture’

The fifth sub-theme related to negative workplace culture which was mentioned by 35 midwives. The lack of acknowledgement from the organisation, not feeling valued for the work they do, feeling under supported or uncared for by their managers, and a lack of adequate communication all contributed to low staff morale and not feeling heard.

*“Management don’t seem to care about the problems staff are experiencing as they are not on ‘the coal face of day to day running of the shift’. There seems to be a ‘just get on with it’ mentality from senior management which is inappropriate and unjust* (ID 297, aged 40, 11 years’ experience, permanent).

Some found the team they worked with difficult, that there was a lack of support and a lack of teamwork. Issues with senior staff was a common concern, feeling bullied or that they couldn’t approach them for assistance.

*“…being scared to ask an AUM [Associate Unit Manager] for help because you know she will push you off to the medical team or know she will yell at you in front of patients is not on.”* (ID 358, aged 28, 7 years’ experience, casual).

Midwives described a sense of frustration, feeling midwifery as a profession was not respected or that midwives were disempowered through the organisation, having disrespectful relationships with medical colleagues, or experiencing occupational violence, ageism or being devalued for their experience.

*“Less recognition for knowledge and a feeling that midwifery is becoming more a profession of obstetric nursing than true midwifery or the midwifery I felt I learned about and started with at the beginning”* (ID 587, aged 45, 16 years’ experience, casual).

#### 'Remuneration not matching effort’

In the final sub-theme, nine midwives felt the remuneration for their work did not match the effort that was required. They believed that there were other careers where they could be paid better and that have less stress. Many were frustrated that they were underpaid and undervalued as midwives.

*“Feel like I could work with less stress and physical tiredness in another profession and earn more money at the same time”* (ID 259, aged 24, 2 years’ experience, permanent).

*“I feel tremendously underpaid and not valued…There are no incentives to stay in this health job and the responsibilities are way too high”* (ID 485, aged 39, 17 years’ experience, permanent).

After initial analysis of the open-ended data, the themes were explored by the age of the midwives. Consistent with responses to the quantitative data, those aged ≤ 30 years more commonly cited the impact of shift work and night duty, the physical and emotional toll from their job and remuneration not matching the hard work and effort as their main reasons for leaving the profession. Those aged 31–45 years were more likely to talk about the impact of shift work and night duty on their work/life balance in relation to family. These themes were less frequent among those over 45 years. Regardless of age, all groups reported heavy workloads, a lack of being able to provide quality care (leading to job dissatisfaction), and a negative workplace culture as factors that influenced them to think about leaving the profession.

## Discussion

This study found a high prevalence of intention to leave the midwifery profession among midwives from two study sites in Melbourne, Australia. The main reasons for leaving for all midwives were not wanting to work shift work, feeling worn out, and experiencing work-related stress. Work-related burnout, poor job satisfaction and a high intention to leave the workplace were all associated with a high intention to leave the profession. Age did not impact intention to leave of itself, but younger and older midwives reported different reasons for considering leaving the profession. These findings will be used to discuss potential strategies to assist with retention and attrition of midwives in the midwifery profession.

### Prevalence of intention to leave the profession

In the 12 months prior to the study, 52% of midwives had thought about leaving the profession, a figure similar to the other Australian studies that reported the prevalence as 43% [12] and 46% [11], which suggests that there may be retention issue in the midwifery profession throughout Australia. One in five midwives in the study had a high intention to leave. As noted in the other studies [11, 12], this level of intention to leave is concerning, and could have serious ramifications for the sustainability of maternity care provision. A lower percentage of midwives (12%) in this study planned to leave the profession in the next five years, compared to other Australian studies (which had rates 24% [11] and 29% [12]). As the overall cohort of midwives in this study are younger (average age 38) than the average age of midwives in the other studies (average ages 47 [11] and 46 [12]), this may be why there are lower percentages planning to leave in the near future.

### Age and intention to leave

In this study age was not associated with a high intention to leave the profession in either the univariate or multivariable analysis, suggesting that younger midwives may be just as likely to leave as older midwives in this population (despite the impact of retirement). This has differed from the two previous Australian studies, one that found older age was associated with a higher intention to leave [11] and one finding midwives under 40 were most likely to leave [12]. This may be related to difference in age cohorts between the studies as previously mentioned.

### Reasons for leaving the profession and factors associated with high intention to leave

The reasons found for potentially exiting the profession and factors that were associated with high intention to leave the profession were closely related in both the quantitative and open-ended responses which together enhance the main (quantitative) findings. Consideration of all the data outlined in the results provides four key areas to focus on to promote a sustainable midwifery profession. The key areas are ‘*shift work’*, ‘*physical and emotional fatigue/burnout and work-related stress*’, ‘*dissatisfaction associated with midwifery’* and ‘*workplace culture’*.

### Shift work

The impact of working shifts and wanting set shifts featured highly in younger midwives’ reasons for thinking about leaving the profession. A study conducted with early career midwives in the United Kingdom also reported roster issues and the impact of shift work causing poor physical and emotional health and had a negative impact on their relationships and social lives [27]. Previous research has shown that midwives who are affected by work-life conflict have an increased intention to leave [28], and several studies have shown that the impact of being a midwife on work/life balance and family commitments are reasons midwives leave the profession [11, 13, 15]. Addressing the impact of shift work has important implications for midwifery workforce planning. At this stage in Australia, there are very limited options to practice as a midwife outside of shift work. Introduction of models of care or opportunities for midwives to have non-shift work options might be crucial in increasing the longevity of younger midwives in the profession. Other possible strategies include flexible rostering practices and the introduction of more options for set shifts. Consideration for those who are unable to work shift work or need set shifts at various stages of their career may address retention for some midwives.

### Physical and emotional fatigue/burnout and work-related stress

Experiencing work-related stress, being worn out, facing increased workloads and the emotional and physical toll of working in midwifery were all reasons or themes for potentially exiting the profession. Harvie et al. [12] reported Australian midwives ‘being at breaking point’ (p.e589) and wanting to leave the profession due to stress, feeling overwhelmed, being under pressure, and feeling anxious, burnt out and exhausted. Previous work by Hammig [29] has found that physical, emotional, and mental workload and job stress are strongly associated with intention to leave among healthcare professionals.

In this study we found that experiencing work-related burnout was associated with four-fold increased odds of having a high intention to leave the profession. This is in line with other studies that have found a link between burnout and increased intention to leave the profession [13, 14]. Given this, it is important to think about what factors might help decrease burnout in midwives, and one such factor appears to be working in some types of midwifery continuity of care models. Several Australian studies have found working in continuity models reduces burnout for midwives in comparison to those working in non-continuity models [16, 30, 31]. Despite strong evidence of the positive outcomes associated with continuity of care models (for both women [32] and midwives [16, 30, 31]), in 2022, only 6% of Australian midwives worked in a continuity model in maternity services [33]. Introducing more opportunities to work in continuity of care models is one strategy that may reduce burnout and thus reduce intention to leave.

Despite all the challenges noted, midwives in our study spoke of their love of midwifery. This would indicate that it is not midwifery, rather the working conditions that midwives find themselves exposed to that are influencing their intention to leave the profession. This is reflected in Geraghty et al. [34] study of midwife workplace stress, where the authors described ‘midwifery is stressful but it’s not the job itself’ as one of the major themes. Harvie et al. [12] found that despite the reasons cited for leaving the profession, Australian midwives also loved being midwives and they were passionate about making a difference and being with women. Reducing workloads and improving working conditions (as mentioned by midwives in open-ended responses) may allow midwives to provide better quality of care and thus increase job satisfaction.

### Dissatisfaction associated with midwifery

For those who had low job satisfaction, there was three times higher odds of having a high intention to leave the profession. More than a third of midwives in this study stated ‘disillusionment with midwifery’ as a reason to leave the profession and this was a finding that affected all age groups in the study. Many other studies [12, 14, 15, 35] reflect this finding; dissatisfaction with the organisation of midwifery is a strong and consistent theme in studies exploring intention to leave, and studies [13, 36, 37] that have explored the views of midwives who have left the profession. Studies with other health professionals have noted the importance of job satisfaction on the sustainability of those professions [38–40]. Studies that focus on factors affecting job satisfaction with midwives have found that working in continuity of care models [16, 30] and receiving adequate support and acknowledgement from managers and organisations [19, 41] may assist in improving job satisfaction. Addressing midwifery relationships (such as support from colleagues and managers), and midwifery practice (increasing autonomy and continuity of care models) are potential strategies that would assist with midwifery attrition across all cohorts.

Half of the midwives who were aged ≤ 30 years in this study also felt they could earn more money in other careers and/or experience less ‘heavy’ work for better remuneration. A qualitative study of early career midwives in Australia (six to seven years post qualification) found that remuneration was a consistent theme, that early-career midwives felt that wages were unequal to midwifery responsibilities, and that the lack of appropriate pay was an indicator of lack of acknowledgement and respect of the profession [42]. Midwives in our study who were aged 30 or younger felt there was other better paid sectors for which they could easily retrain, and where they may be more able to work at increased hours. Thus, in their opinion, this would make them financially better off with less work-related stress. Strong consideration should be given to increasing remuneration for midwives to reflect the work they do, and to decrease attrition and reduce competition from other sectors.

### Workplace culture

The strongest factor associated with high intention to leave the *profession* was a high intention to leave the *workplace*, which increased the odds of leaving the profession by almost 14-fold. While it could be argued the intention to leave the workplace and profession are likely to be correlated, other studies have found a link between intention to leave the workplace and increased intention to leave the profession, particularly among younger midwives [28]. The experience of unfavourable working conditions combined with poor workplace culture may push midwives and nurses out of both the workplace and the profession [12, 43]. In this study, regardless of age, many midwives reported a negative workplace culture as a reason to leave the profession. Addressing aspects of poor workplace culture such as providing mentoring (to improve relationships between colleagues) [42], frequent group meetings with facilitated exercises to build respect, collaboration and trust building between different professions providing maternity care [44], and training and managerial supportive policies to manage workplace bullying and occupational violence [45] are strategies that could improve workplace culture.

### Strengths and limitations

There was a relatively high response rate from midwives in this study (64% overall and 95% from site one permanent midwives, that made up 63% of the sample), which increases the validity of the results. Reporting intention to leave by how frequently it was thought about also allowed a nuanced understanding of factors affecting *high* intention to leave as opposed to *any* intention to leave, and the use of validated scales to measure outcomes such as job satisfaction and burnout increase the validity and reliability of these findings. Measuring reasons for leaving the profession was a ‘tick all that applies’ question – this may lead to undue emphasis on some of the reasons indicated, as midwives could not indicate how important reasons were to them, all reasons were given equal value, however the open-ended data strongly reflects the results of the quantitative data, which strengthens the findings. This study also benefits from including midwives from two different workplaces and both permanently and casually employed midwives, potentially increasing the generalisability of the results. However there was a difference in response rate between the two sites which may affect the generalisability of the results. The demographics of site two showed an older, more experienced cohort who were more likely to have had children compared to site one and a lower percentage of this group responded to the survey. We don’t know the demographics of those who did not respond and whether that impacted the response rate or there may have been other factors that we cannot account for as to why less midwives responded to the survey from site two. At both sites there was high engagement with midwives about the survey in an effort to make sure potential respondents knew about the survey and had the chance to complete it if they wished.

The cohort overall was younger (the average age was 38) that the average age of an Australian midwife, which is 48 [8], and worked in metropolitan Melbourne, both factors which could potentially limit the generalisability to the whole population of Australian midwives. As this is a cross-sectional study, we present associations, and cannot show causality. The study was conducted prior to the COVID-19 pandemic, so midwives’ level of burnout, job satisfaction and intention to leave was not impacted by the effects of the COVID-19 pandemic. We don’t know if these outcomes would have been worse if measured during or after the pandemic, or if there might be higher intention to leave.

## Conclusion

This study found a high percentage of midwives intending to leave the profession. Those who were at highest risk of leaving were experiencing work-related burnout, poor job satisfaction and strongly thinking of leaving their workplace. Reasons for considering leaving included not wanting to do shift work, feeling worn out, and experiencing work-related stress. Strategies, such as offering non-shift work midwifery care options, flexible rostering, options for set shifts, increasing renumeration, and addressing issues such as work-related stress, dissatisfaction with midwifery and negative workplace cultures may help with retention of midwives. As this study was conducted pre-pandemic, further exploration of the wellbeing and work intents of midwives in the current post-pandemic context is important to ensure any strategies that are implemented are relevant, and to ensure we have a current picture of the prevalence of intention to leave the profession among midwives.

## Supplementary Information

Supplementary Material 1.

Supplementary Material 2.

Supplementary Material 3.

## Data Availability

No datasets were generated or analysed during the current study.
