# Supplementary material for: I love my job. But it’s physically, mentally, and emotionally draining”: a cross-sectional survey exploring midwives’ intentions of leaving the profession in Melbourne, Australia
Source: BMC Health Serv Res. 2024 Nov 26;24:1471. doi: 10.1186/s12913-024-11863-7 (PMC11590212; doi:10.1186/s12913-024-11863-7)
Supplement: Supplementary file 1 — Supplementary Material 1. [file 12913_2024_11863_MOESM1_ESM.docx]

**Table S1: Intention to leave the midwifery profession studies**

| **Study, country, year conducted, participants** | **Survey design, distribution** | **Population, response rate** | **Age** | **Years of experience** | **Intention to leave (ITL)** | **Factors associated and reasons for leaving** |
| --- | --- | --- | --- | --- | --- | --- |
| Pugh et al. (10)  Australia  2010  n=712 | Cross-sectional, postal | Midwives currently employed in public and private maternity services in Western Australia (n=3,408)  21% response rate | 63% ≥45 years  Avg. age 47 | 66% ≥10 years  Avg. 16 years | 46% ITL  24% ITL next 5 years | Older age  Main reason for 59% was retirement  20% of potential leavers aged <55 years  Those aged <55 years reasons for leaving:   - work-life balance - career change - family commitments - working conditions - rostering |
| Harvie et al. (11)  Australia  2014  n=1037 | Cross-sectional, online | All members of Australian College of Midwives (n=4600) and survey was distributed via professional networks  Response rate not stated | 74% ≥40 years  Avg. age 46 | 64% ≥10 years  Avg. 16 years | 43% ITL in 6 months prior to study  27% ITL next 5 years | Reasons   - dissatisfaction with the organisation of midwifery care, - dissatisfaction with the role of a midwife - family commitments - fear of litigation - poor pay - planned career change - ill health - planned retirement   Midwives with children less likely to consider leaving than those without children  Midwives aged < 40 most likely to leave due to dissatisfaction |
| Hunter et al. (13)  United Kingdom  2017  n=1997 | Cross-sectional, online | All full midwife members of Royal College of Midwives (n=31898)  Response rate 16% | 68% ≥40 years  Avg. age 47 | Median 16 years | 66% ITL in the 6 months prior to study | Reasons   - staff shortages - lack of quality care provided - dissatisfaction with organisation of midwifery care |
| Stoll and Gallagher (12) Canada  2017  n=158 | Cross-sectional, online | All currently practising registered midwives in British Columbia, Alberta, and Ontario (n=1100)  Response rate 14% | Avg. age 41 | Avg. 9 years | 67% ITL in 12 months prior study  29% ITL next five years | Reasons   - negative impact of on-call schedule on their lives - concerns about their mental and physical health |
| Feijen-de Jong et al (14)  The Netherlands  2019  n=726 | Cross-sectional, postal and online | Currently practising midwives working in community care  Two step recruitment  1) A random sample of practising midwives registered in a research database which was supplemented with all midwives qualified < 3 years from that database were sent an invitation (n=1241)  2) All Royal Dutch Organisation of Midwives members sent an email  (n=not stated)  Response rate not stated | 40% ≥40 years | 54% ≥10 years | 34% ITL in 6 months prior to study | Reasons   - dissatisfaction with organisation of midwifery care - family commitments   Midwives with >3 years’ experience were more likely leave due to dissatisfaction with the organisation of midwifery care and family commitments |
