# Supplementary material for: I love my job. But it’s physically, mentally, and emotionally draining”: a cross-sectional survey exploring midwives’ intentions of leaving the profession in Melbourne, Australia
Source: BMC Health Serv Res. 2024 Nov 26;24:1471. doi: 10.1186/s12913-024-11863-7 (PMC11590212; doi:10.1186/s12913-024-11863-7)
Supplement: Supplementary file 2 — Supplementary Material 2. [file 12913_2024_11863_MOESM2_ESM.docx]

**Table S2: Univariate and multivariable analysis of intention to leave the profession (multivariable n=176)**

|  | | | n^a^ | % | OR | p value | 95% CI | Adj. OR | Adj. p value^b^ | 95% CI |
| --- | --- | --- | --- | --- | --- | --- | --- | --- | --- | --- |
| Age | | | | | | | | | | |
|  | | ≤ 30 years | 28/131 | 21 | 1 |  |  |  |  |  |
|  | | 31-45 years | 15/91 | 16 | 0.73 | 0.37 | 0.36, 1.45 |  |  |  |
|  | | 46-54 years | 9/43 | 21 | 0.97 | 0.95 | 0.42, 2.27 |  |  |  |
|  | | > 55 years | 6/34 | 18 | 0.79 | 0.63 | 0.30, 2.09 |  |  |  |
| Marital status | | | | | | | | | | |
|  | | In relationship | 39/200 | 20 | 1 |  |  |  |  |  |
|  | | Single | 18/93 | 19 | 0.99 | 0.98 | 0.53, 1.85 |  |  |  |
| Children | | | | | | | | | | |
|  | Yes | | 24/134 | 18 | 1 |  |  |  |  |  |
|  | No | | 36/171 | 21 | 1.22 | 0.49 | 0.69, 2.17 |  |  |  |
| Carer for someone other than children | | | | | | | | | | |
|  | No (ref) | | 55/288 | 19 | 1 |  |  |  |  |  |
|  | Yes | | 6/20 | 25 | 1.41 | 0.52 | 0.49, 4.05 |  |  |  |
| Years post qualification | | | | | | | | | | |
|  | ≤5 years | | 25/145 | 17 | 1 |  |  |  |  |  |
|  | 6-10 years | | 10/63 | 16 | 0.91 | 0.81 | 0.41, 2.02 |  |  |  |
|  | >10 years | | 22/92 | 24 | 1.51 | 0.21 | 0.79, 2.87 |  |  |  |
| Work site | | | | | | | | | | |
|  | Site one | | 56/281 | 20 | 1 |  |  |  |  |  |
|  | Site two | | 3/25 | 12 | 0.55 | 0.34 | 0.16, 1.90 |  |  |  |
|  | Both | | 1/2 | 50 | 4.02 | 0.33 | 0.25, 65.23 |  |  |  |
| Work hours | | | | | | | | | | |
|  | Casual | | 9/42 | 21 | 1 |  |  |  |  |  |
|  | Part-time | | 35/189 | 19 | 0.83 | 0.67 | 0.37, 1.90 |  |  |  |
|  | Full-time | | 15/75 | 20 | 0.92 | 0.85 | 0.36, 2.32 |  |  |  |
| Type of work hours | | | | | | | | | | |
|  | Non shift worker | | 13/62 | 21 | 1 |  |  |  |  |  |
|  | Shift worker | | 47/246 | 19 | 0.89 | 0.74 | 0.45, 1.77 |  |  |  |
| Work area | | | | | | | | | | |
|  | Work most or all areas | | 50/268 | 19 | 1 |  |  |  |  |  |
|  | Works in one area | | 10/37 | 27 | 1.61 | 0.23 | 0.73, 3.55 |  |  |  |
| Work elsewhere | | | | | | | | | | |
|  | No | | 52/258 | 20 | 1 |  |  |  |  |  |
|  | Yes | | 9/51 | 18 | 0.85 | 0.68 | 0.39, 1.85 |  |  |  |
| Personal burnout | | | | | | | | | | |
|  | | No | 4/80 | 5 | 1 |  |  | 1 |  |  |
|  | | Yes | 38/149 | 26 | 6.50 | 0.001 | 2.23, 18.98 | n/a |  |  |
| Work-related burnout | | | | | | | | | | |
|  | | No | 8/120 | 7 | 1 |  |  | 1 |  |  |
|  | | Yes | 34/108 | 31 | 6.43 | <0.0001 | 2.82, 14.67 | 4.03 | 0.02 | 1.20, 13.53 |
| Client-related burnout | | | | | | | | | | |
|  | | No | 32/205 | 16 |  |  |  | 1 |  |  |
|  | | Yes | 10/20 | 50 | 5.41 | 0.001 | 2.08, 14.04 | n/a |  |  |
| Negative attitude to professional satisfaction | | | | | | | | | | |
|  | | No | 20/173 | 12 | 1 |  |  | 1 |  |  |
|  | | Yes | 16/32 | 50 | 7.65 | <0.0001 | 3.32, 17.63 | 3.08 | 0.03 | 1.09, 8.68 |
| Negative attitude to professional support | | | | | | | | | | |
|  | | No | 11/101 | 11 | 1 |  |  | 1 |  |  |
|  | | Yes | 30/110 | 27 | 3.07 | 0.004 | 1.44, 6.52 | n/a |  |  |
| Negative attitude to client interaction | | | | | | | | | | |
|  | | No | 16/110 | 15 | 1 |  |  | 1 |  |  |
|  | | Yes | 23/95 | 24 | 1.88 | 0.08 | 0.92, 3.81 | n/a |  |  |
| Negative attitude to professional development | | | | | | | | | | |
|  | | No | 28/168 | 17 | 1 |  |  | 1 |  |  |
|  | | Yes | 14/46 | 30 | 2.19 | 0.04 | 1.04, 4.62 | n/a |  |  |
| Able to take regular breaks | | | | | | | | | | |
|  | | Yes | 24/158 | 15 | 1 |  |  | 1 |  |  |
|  | | No | 17/67 | 25 | 1.90 | 0.07 | 0.94, 3.83 | n/a |  |  |
| Consider the skill mix to be unsafe | | | | | | | | | | |
|  | | No | 14/77 | 18 | 1 |  |  |  |  |  |
|  | | Yes | 30/161 | 19 | 1.03 | 0.93 | 0.51, 2.08 |  |  |  |
| Adequate acknowledgement from organisation | | | | | | | | | | |
|  | | Yes | 6/78 | 8 | 1 |  |  | 1 |  |  |
|  | | No | 36/138 | 26 | 4.24 | 0.002 | 1.70, 10.58 | n/a |  |  |
| Need more support | | | | | | | | | | |
|  | | No | 20/135 | 15 | 1 |  |  | 1 |  |  |
|  | | Yes | 22/97 | 23 | 1.69 | 0.13 | 0.86, 3.30 | n/a |  |  |
| Worried about approaching senior staff | | | | | | | | | | |
|  | | No | 32/167 | 19 | 1 |  |  |  |  |  |
|  | | Yes | 9/63 | 14 | 0.70 | 0.39 | 0.31, 1.57 |  |  |  |
| High/low intention to leave the workplace | | | | | | | | | | |
|  | | Low intention to leave | 9/187 | 5 | 1 |  |  | 1 |  |  |
|  | | High intention to leave | 50/118 | 42 | 14.54 | <0.0001 | 6.78, 31.18 | 13.92 | <0.0001 | 3.83, 50.52 |

OR Odds Ratio.

Adj. Adjusted

^a^ The number of midwives in each category who had a high intention to leave profession (e.g., 28 out of 131 midwives aged ≤30 had a high intention to leave the profession)

^b^ includes only those factors where an association was maintained in the multivariate regression.
