# Supplementary material for: I love my job. But it’s physically, mentally, and emotionally draining”: a cross-sectional survey exploring midwives’ intentions of leaving the profession in Melbourne, Australia
Source: BMC Health Serv Res. 2024 Nov 26;24:1471. doi: 10.1186/s12913-024-11863-7 (PMC11590212; doi:10.1186/s12913-024-11863-7)
Supplement: Supplementary file 3 — Supplementary Material 3. [file 12913_2024_11863_MOESM3_ESM.docx]

**Supplementary File 3 – The EXPert survey**

| **Section 1: Questions about you.** | | | | | | | |  |
| --- | --- | --- | --- | --- | --- | --- | --- | --- |
| These questions relate to your career in nursing and/or midwifery and some general questions about yourself | | | | | | | |  |
| **1.01** | **Are you a mothercraft nurse?** | | | | | | |  |
|  | **_1_** | Yes *(go to question 1.04)* | | | | | |  |
|  | **_2_** | No *(go to question 1.02)* | | | | | |  |
|  | **_3_** | I was, but no longer registered as a mothercraft nurse | | | | | |  |
| **1.02** | **Are you an enrolled nurse?** | | | | | | |  |
|  | **_1_** | Yes *(go to question 1.04)* | | | | | |  |
|  | **_2_** | No *(go to question 1.03)* | | | | | |  |
|  | **_3_** | I was, but no longer registered as an enrolled nurse | | | | | |  |
| **1.03** | **Are you a registered nurse?** | | | | | | |  |
|  | **_1_** | Yes | | | | | |  |
|  | **_2_** | No *(go to question 1.09)* | | | | | |  |
|  | **_3_** | I was, but no longer registered as a nurse | | | | | |  |
| **1.04** | **What year did you first register as a nurse?** | | | | | | |  |
|  |  | | | | | | |  |
| **1.05** | **How many years have you practiced as a nurse? (round to the nearest year)**  If you are not currently working as a nurse although you are still registered then indicate that in the number of years you have practiced as a nurse, i.e. only include the years you actually practiced | | | | | | |  |
|  | years | | | | | | |  |
| **1.06** | **Did you complete your education/training to become registered/enrolled/mothercraft nurse in Australia?** | | | | | | |  |
|  | **_1_** | | Yes *(go to question 1.08)* | | | | |  |
|  | **_2_** | | No | | | | |  |
| **1.07** | **Which country did you complete your education/training as a nurse?** | | | | | | |  |
|  |  | | | | | | |  |
| **1.07b** | **Please describe what education/training you did to become a registered/enrolled/mothercraft nurse** | | | | | | |  |
|  |  | | | | | | |  |
|  | **Please now go to question 1.09** | | | | | | |  |
| **1.08** | **How did you first qualify to become a nurse?** | | | | | | |  |
|  | **_1_** | TAFE (Technical and Further Education) course | | | | | |  |
|  | **_2_** | University diploma | | | | | |  |
|  | **_3_** | University degree | | | | | |  |
|  | **_4_** | Hospital (only) program | | | | | |  |
| **1.09** | **Are you a registered midwife?** | | | | | | |  |
|  | **_1_** | Yes | | | | | |  |
|  | **_2_** | No *(go to question 1.15)* | | | | | |  |
|  | **_3_** | I was, but no longer registered as a midwife | | | | | |  |
| **1.10** | **What year did you first register as a midwife?** | | | | | | |  |
|  |  | | | | | | |  |
| **1.11** | **How many years have you practiced as a midwife? (round to the nearest year)**  If you are not currently working as a midwife although you are still registered then indicate that in the number of years you have practiced as a midwife, i.e. only include the years you actually practiced | | | | | | |  |
|  | years | | | | | | |  |
| **1.12** | **Did you complete your education/training to become registered midwife in Australia?** | | | | | | |  |
|  | **_1_** | | Yes *(go to question 1.15)* | | | | |  |
|  | **_2_** | | No | | | | |  |
| **1.13** | **Which country did you complete your education/training as a registered midwife?** | | | | | | |  |
|  |  | | | | | | |  |
| **1.13b** | **Please describe what education/training you did to become a registered midwife** | | | | | | |  |
|  |  | | | | | | |  |
| **1.14** | **How did you first qualify to become a registered midwife?** | | | | | | |  |
|  | **_1_** | | University degree (undergraduate) | | | | |  |
|  | **_2_** | University degree (postgraduate) | | | | | |  |
|  | **_3_** | Hospital (only) program | | | | | |  |
| **1.15** | **Since your initial registration as a nurse and/or midwife have you had any breaks in practice? (e.g. maternity leave, leave without pay, NOT including annual or long service leave)** | | | | | | |  |
|  | **_1_** | Yes | | | |  |  |  |
|  | **_2_** | No *(go to question 1.17)* | | | |  |  |  |
| **1.16** | **What is the TOTAL amount of time that you have had away from nursing and/or midwifery? (e.g. 3 years TOTAL of maternity leave or 12 months of leave without pay, NOT including annual or long service leave) – put 0 in years if you have been away for <12months and indicate number of months** | | | | |  |  |  |
|  | years and months | | | | |  |  |  |
| **1.17** | **What other qualifications do you hold that are related to nursing and midwifery? (tick all that apply)** | | | | | | |  |
|  | **_1_** | None | | | | | |  |
|  | **_2_** | Certificate | | | | | |  |
|  | **_3_** | Diploma | | | | | |  |
|  | **_4_** | Degree | | | | | |  |
|  | **_5_** | Graduate diploma | | | | | |  |
|  | **_6_** | Master’s degree | | | | | |  |
|  | **_7_** | PhD | | | | | |  |
|  | **_8_** | Currently enrolled in a higher degree- please describe: | | | | | |  |
|  | **_9_** | Other- please describe: | | | | | |  |
| **1.18** | **What other qualifications do you hold that are not related to nursing and midwifery? (tick all that apply)** | | | | | | |  |
|  | **_1_** | None | | | | | |  |
|  | **_2_** | Certificate | | | | | |  |
|  | **_3_** | Diploma | | | | | |  |
|  | **_4_** | Degree | | | | | |  |
|  | **_5_** | Graduate diploma | | | | | |  |
|  | **_6_** | Master’s degree | | | | | |  |
|  | **_7_** | PhD | | | | | |  |
|  | **_8_** | Currently enrolled in a higher degree - please describe: | | | | | |  |
|  | **_9_** | Other - please describe: | | | | | |  |
| **1.19** | **What is your age?** | | | | | | |  |
|  | years | | | | | | |  |
| **1.20** | **Would you describe your gender as;** | | | | | | |  |
|  | **_1_** | Female | | | | | |  |
|  | **_2_** | Male | | | | | |  |
|  | **_3_** | Other | | | | | |  |
|  | **_4_** | Prefer not to say | | | | | |  |
| **1.21** | **Were you born in Australia?** | | | | | | |  |
|  | **_1_** | Yes *(go to question 1.23)* | | | | | |  |
|  | **_2_** | No | | | | | |  |
| **1.22a** | **What is your country of birth?** | | | | | | |  |
|  |  | | | | | | |  |
| **1.22b** | **How long have you lived in Australia?** | | | | | | |  |
|  | years | | | | | | |  |
| **1.23** | **Would you describe your marital status as;** | | | | | | |  |
|  | **_1_** | Single | | | | | |  |
|  | **_2_** | De facto | | | | | |  |
|  | **_3_** | Married | | | | | |  |
|  | **_4_** | Prefer not to say | | | | | |  |
| **1.24** | **Do you have children?** | | | | | | |  |
|  | **_1_** | Yes | | | | | |  |
|  | **_2_** | No *(go to question 1.26)* | | | | | |  |
| **1.25** | **What age are your children? (tick all that apply)** | | | | | | |  |
|  | **_1_** | Younger than primary school | | | | | |  |
|  | **_2_** | Primary school age | | | | | |  |
|  | **_3_** | Secondary school age | | | | | |  |
|  | **_4_** | Older than secondary school age | | | | | |  |
| **1.26** | **Are you a carer for another person/other people (not including children in this instance)? (e.g. parents, siblings, partner)** | | | | | | |  |
|  | **_1_** | Yes | | | | | |  |
|  | **_2_** | No | | | | | |  |
| **1.27** | **In which [organisation] campus do you work?** | | | | | | |  |
|  | **_1_** | Site one | | | | | |  |
|  | **_2_** | Site two | | | | | |  |
|  | **_3_** | Both | | | | | |  |
| **1.28** | **Have you been working at the [organisation] for more than a year?** | | | | | | |  |
|  | **_1_** | Yes *(go to question 1.30)* | | | | | |  |
|  | **_2_** | No *(go to question 1.29)* | | | | | |  |
| **1.29** | **How long have you been working as a nurse/midwife at the [organisation]? (round to the nearest month)** | | | | | | |  |
|  | months *now go to question 1.31* | | | | | | |  |
| **1.30** | **How long have you been working as a nurse/midwife at the [organisation]? (round to the nearest year)** | | | | | | |  |
|  | years | | | | | | |  |
| **1.31** | **Have you previously worked anywhere else apart from the [organisation] as a nurse and/or midwife?** | | | | | | |  |
|  | **_1_** | Yes | | | | | |  |
|  | **_2_** | No *(go to question 1.33)* | | | | | |  |
| **1.32** | **Please tick places that you have previously worked apart from the [organisation] (tick all that apply)** | | | | | | |  |
|  | **_1_** | | Another tertiary hospital | | | |  |  |
|  | **_2_** | | Non-tertiary public metropolitan hospital | | | |  |  |
|  | **_3_** | | Regional hospital | | | |  |  |
|  | **_4_** | | Rural hospital | | | |  |  |
|  | **_5_** | | Remote hospital or service | | | |  |  |
|  | **_6_** | | Private hospital | | | |  |  |
|  | **_7_** | | Agency | | | |  |  |
|  | **_8_** | | Free standing birth centre | | | |  |  |
|  | **_9_** | | As a midwife in private practice | | | |  |  |
|  | **_10_** | | Community health clinic/GP clinic | | | |  |  |
|  | **_11_** | | Maternal and Child Health Centre | | | |  |  |
|  | **_12_** | | Midwife with a private obstetrician | | | |  |  |
|  | **_13_** | | Interstate | | | |  |  |
|  | **_14_** | | Overseas | | | | |  |
|  | **_15_** | | Other - please describe. | | | | |  |
| **1.33** | **What hours do you work at the [organisation]?** | | | | | | |  |
|  | **_1_** | Casual | | | | | |  |
|  | **_2_** | Part time *(go to question 1.35)* | | | | | |  |
|  | **_3_** | Full time *(go to question 1.36)* | | | | | |  |
| **1.34** | **How many casual hours do you work on average each fortnight at the [organisation]?** | | | | | | |  |
|  | hours *now go to question 1.36* | | | | | | |  |
| **1.35** | **How many part time hours do you work on average each fortnight at the [organisation]?** | | | | | | |  |
|  | hours | | | | | | |  |
| **1.36** | **Which of the following best describes the shift pattern of your work at the [organisation]? (tick all that apply)** | | | | | | |  |
|  | **_1_** | Monday to Friday | | | | | |  |
|  | **_2_** | Rotating shift work | | | | | |  |
|  | **_3_** | Permanent night shift | | | | | |  |
|  | **_4_** | On-call | | | | | |  |
|  | **_5_** | Other- please describe: | | | | | |  |
|  |  |  | | | | | |  |
| **1.37** | **In what capacity are you currently employed at the [organisation]? (tick all that apply)** | | | | | | |  |
|  | **_1_** | Mothercraft nurse | | | | | |  |
|  | **_2_** | Enrolled nurse | | | | | |  |
|  | **_3_** | Graduate nurse (in first 12 months of practice) | | | | | |  |
|  | **_4_** | Graduate midwife (in first 12 months of practice) | | | | | |  |
|  | **_5_** | Graduate nurse and midwife (in first 12 months of practice) | | | | | |  |
|  | **_6_** | Registered nurse | | | | | |  |
|  | **_7_** | Registered midwife | | | | | |  |
|  | **_8_** | Registered nurse and midwife | | | | | |  |
|  | **_9_** | Clinical nurse specialist | | | | | |  |
|  | **_10_** | Clinical midwife specialist | | | | | |  |
|  | **_11_** | Caseload midwife | | | | | |  |
|  | **_12_** | Diabetes educator | | | | | |  |
|  | **_13_** | Lactation consultant | | | | | |  |
|  | **_14_** | Research nurse and/or midwife | | | | | |  |
|  | **_15_** | Clinic co-ordinator | | | | | |  |
|  | **_16_** | Clinical support nurse | | | | | |  |
|  | **_17_** | Clinical support midwife | | | | | |  |
|  | **_18_** | Clinical educator | | | | | |  |
|  | **_19_** | Care manager | | | | | |  |
|  | **_20_** | Nurse practitioner | | | | | |  |
|  | **_21_** | Associate unit manager | | | | | |  |
|  | **_22_** | Clinical nurse consultant/ Clinical midwife consultant | | | | | |  |
|  | **_23_** | Team leader/ Unit manager | | | | | |  |
|  | **_24_** | Senior leadership team (e.g. Director or part of Executive) | | | | | |  |
|  | **_25_** | Other - please describe: | | | | | |  |
|  |  |  | | | | | |  |
| **1.37b** | **If you have more than one role that you are employed in at the [organisation], please indicate which is your MAIN role? (please choose one option from question 1.37 and write down the number)** | | | | | | |  |
|  |  | | | | | | |  |
| **1.38** | **Which areas of the hospital do you currently work in? (tick all that apply)** | | | | | | |  |
|  | **_1_** | After Hours Manager | | | | | |  |
|  | **_2_** | Anaesthetics/Pain Services | | | | | |  |
|  | **_3_** | Breastfeeding Services | | | | | |  |
|  | **_4_** | Childbirth Education | | | | | |  |
|  | **_5_** | Clinical Education | | | | | |  |
|  | **_6_** | Complex Care Unit | | | | | |  |
|  | **_7_** | Early Pregnancy Assessment Service | | | | | |  |
|  | **_8_** | Executive | | | | | |  |
|  | **_9_** | Infection Control | | | | | |  |
|  | **_10_** | Maternity Services | | | | | |  |
|  |  | | | **_1_** | Antenatal Clinic (including team clinic co-ordinator) | | |  |
|  |  | | | **_2_** | Antenatal Ward | | |  |
|  |  | | | **_3_** | Assessment Centre | | |  |
|  |  | | | **_4_** | Birth Centre | | |  |
|  |  | | | **_5_** | Postnatal Care In The Home | | |  |
|  |  | | | **_6_** | Postnatal Ward | | |  |
|  |  | | | **_7_** | Pregnancy Day Care | | |  |
|  | **_11_** | Neonatal Services | | | | | |  |
|  |  | | | **_1_** | Special Care Nursery | | |  |
|  |  | | | **_2_** | Neonatal High Dependency Unit | | |  |
|  |  | | | **_3_** | Neonatal Intensive Care | | |  |
|  |  | | | **_4_** | Neonatal Hospital In The Home | | |  |
|  |  | | | **_5_** | ROP clinic | | |  |
|  | **_12_** | Pauline Gandel Imaging Centre | | | | | |  |
|  | **_13_** | Perioperative Services/Surgical Services | | | | | |  |
|  |  | | | **_1_** | Elective Surgery Access Service (Bookings) | | |  |
|  |  | | | **_2_** | Operating Theatres | | |  |
|  |  | | | **_3_** | Day Surgery Unit | | |  |
|  |  | | | **_4_** | Post Anaesthetic Care Unit | | |  |
|  | **_14_** | Reproductive Services | | | | | |  |
|  | **_15_** | Research Unit/Centre | | | | | |  |
|  | **_16_** | Sexual Assault Crisis Line/CASA House | | | | | |  |
|  | **_17_** | Specialist area (e.g. Diabetes Education, Multiple Births, Fetal Management Unit, Reproductive Loss, Mental Health) | | | | | |  |
|  | **_18_** | WHIC ([organisation] Health Information Centre) | | | | | |  |
|  | **_19_** | **[organisation]** Emergency Care | | | | | |  |
|  | **_20_** | **[organisation]** Gynaecology and Cancer Unit | | | | | |  |
|  | **_21_** | **[organisation]** Health Clinics | | | | | |  |
|  | **_22_** | Other - please describe: | | | | | |  |
|  |  |  | | | | | |  |
| **1.39** | **Do you also work as a nurse or midwife somewhere other than the [organisation]?** | | | | | | |  |
|  | **_1_** | Yes | | | | | |  |
|  | **_2_** | No *(go to question 1.41)* | | | | | |  |
| **1.40** | **What is the position that you are employed in elsewhere? (please describe)** | | | | | | |  |
|  |  | | | | | | |  |
| **1.41** | **How long do you plan to continue working at the [organisation]?** | | | | | | |  |
|  | **_1_** | <1 year | | | | | |  |
|  | **_2_** | 1-2 years | | | | | | |
|  | **_3_** | 3-5 years | | | | | | |
|  | **_4_** | 6-10 years | | | | | | |
|  | **_5_** | 11-20 years | | | | | | |
|  | **_6_** | >20 years | | | | | | |
|  | **_7_** | Not sure | | | | | | |
| **1.42** | **In the last 12 months how often have you thought about leaving the [organisation]?** | | | | | | |  |
|  | **_1_** | Never *(go to question 1.44)* | | | | | | |
|  | **_2_** | Occasionally (a couple of times per year) | | | | | | |
|  | **_3_** | Sometimes (monthly) | | | | | | |
|  | **_4_** | Frequently (weekly) | | | | | | |
|  | **_5_** | All the time | | | | | | |
| **1.43** | **What has caused you to think about leaving the [organisation]?** (please describe) | | | | | | |  |
|  |  | | | | | | |  |
| **1.44** | **How long do you plan to continue working in the profession of nursing or midwifery** | | | | | | |  |
|  | **_1_** | <1 year | | | | | |  |
|  | **_2_** | 1-2 years | | | | | | |
|  | **_3_** | 3-5 years | | | | | | |
|  | **_4_** | 6-10 years | | | | | | |
|  | **_5_** | 11-20 years | | | | | | |
|  | **_6_** | >20 years | | | | | | |
|  | **_7_** | Not sure | | | | | | |
| **1.45** | **In the last 12 months have you considered leaving the profession of nursing or midwifery?** | | | | | | |  |
|  | **_1_** | Never *(go to section 2)* | | | | | | |
|  | **_2_** | Occasionally (a couple of times per year) | | | | | | |
|  | **_3_** | Sometimes (monthly) | | | | | | |
|  | **_4_** | Frequently (weekly) | | | | | | |
|  | **_5_** | All the time | | | | | | |
| **1.46** | **What are your reasons for thinking of leaving the profession? (tick all that apply)** | | | | | | |  |
|  | **_1_** | Retirement | | | | | | |
|  | **_2_** | Health concerns (e.g. injury preventing continuing working) | | | | | | |
|  | **_3_** | Disillusionment with nursing and/or midwifery | | | | | | |
|  | **_4_** | Not conducive to raising a family | | | | | | |
|  | **_5_** | Worn out | | | | | | |
|  | **_6_** | Earn more money in other professions | | | | | | |
|  | **_7_** | Wanting to work set days | | | | | | |
|  | **_8_** | Want a career beyond nursing and midwifery | | | | | | |
|  | **_9_** | ‘Nothing left to give’ | | | | | | |
|  | **_10_** | No longer challenged | | | | | | |
|  | **_11_** | Work related stress | | | | | | |
|  | **_12_** | Mental health issues | | | | | | |
|  | **_13_** | Not wanting to work shift work | | | | | | |
|  | **_14_** | Other – please describe | | | | | | |
|  | Please feel free to comment further – this is a really important issue, and we are very keen to have your thoughts | | | | | | | |

| **Section 2: ‘Expertise’** | | | | | | | | |
| --- | --- | --- | --- | --- | --- | --- | --- | --- |
| This next section relates to your views and opinions on the concept of 'expertise' amongst nurses and midwives | | | | | | | | |
| **2.01** | **From the following list of professional attributes what are the THREE MOST important attributes that make a nurse or midwife an ‘expert’?** | | | | | | | |
|  | **_a_** | Shares knowledge with colleagues | | | | | | |
|  | **_b_** | Teaches staff/students | | | | | | |
|  | **_c_** | Updates her/his clinical knowledge and skills regularly | | | | | | |
|  | **_d_** | Admits lack of knowledge | | | | | | |
|  | **_e_** | Is competent in clinical/practical skills | | | | | | |
|  | **_f_** | Good time management skills | | | | | | |
|  | **_g_** | Has a broad knowledge base | | | | | | |
|  | **_h_** | Has many years of experience | | | | | | |
|  | **_i_** | Challenges the traditional practices of the system | | | | | | |
|  | **_j_** | Has been exposed to many complex situations | | | | | | |
| **2.02** | **From the following list of characteristics what are the THREE MOST important characteristics that make a nurse or midwife an ‘expert’?** | | | | | | | |
|  | **_a_** | Communicates well with other staff | | | | | | |
|  | **_b_** | Able to prioritise care | | | | | | |
|  | **_c_** | Is adept at resolving conflicts or concerns | | | | | | |
|  | **_d_** | Prioritises patient experience | | | | | | |
|  | **_e_** | Is a proactive team member | | | | | | |
|  | **_f_** | Is a positive leader and role model | | | | | | |
|  | **_g_** | Is a ‘team player’ | | | | | | |
|  | **_h_** | Communicates well with women/families | | | | | | |
| **2.03** | **From the following list of personal attributes what are the THREE MOST important attributes that make a nurse or midwife an ‘expert’?** | | | | | | | |
|  | **_a_** | Approachable | | | | | | |
|  | **_b_** | Passionate | | | | | | |
|  | **_c_** | Confident | | | | | | |
|  | **_d_** | Has patience | | | | | | |
|  | **_e_** | Intuitive | | | | | | |
|  | **_f_** | Calm | | | | | | |
|  | **_g_** | Adaptable | | | | | | |
|  | **_h_** | Open to change | | | | | | |
|  | **_i_** | Accountable for own actions | | | | | | |
| **2.04** | **Are there any other characteristics that you feel are important in regards to ‘expertise’? Or would you like to tell us more about your choices from the previous questions?** | | | | | | | |
|  |  | | | | | | | |
| **2.05** | **On a scale of ‘1’ to ‘7’, where ‘1’ is a ‘beginner’ and ‘7’ is an ‘expert’ practitioner in nursing or midwifery, where would you rate your current level of ‘expertise’ in your MAIN role at the [organisation]** | | | | | | | |
|  | **Beginner** | |  |  |  |  |  | **Expert** |
|  | 1 | | 2 | 3 | 4 | 5 | 6 | 7 |

| **2.06** | **What assisted you to achieve your current level of ‘expertise’? (tick all that apply)** | |
| --- | --- | --- |
|  | _1_ | Exposure to many complex situations |
|  | _2_ | My ability to meet women’s individual needs |
|  | _3_ | Further education e.g. certificate/degree/masters/PhD |
|  | _4_ | My family/personal relationships have supported me in furthering my career |
|  | _5_ | Confidence in my skills/knowledge |
|  | _6_ | My years of experience |
|  | _7_ | My managers support in furthering my career |
|  | _8_ | Excellent preceptor/role model/mentor |
|  | _9_ | I communicate well with others |
|  | _10_ | I work well within a team |
|  | _11_ | When I encounter problems at work, I find them easy to solve |
|  | _12_ | If I do not know something, I educate myself about it |
|  | _13_ | Passionate about my work |
|  | _14_ | The **[organisation]** has given me many opportunities to develop ‘expertise’ |
|  | _15_ | Working in other places |
|  | _16_ | I am a good leader |
|  | _17_ | I am approachable |
|  | _18_ | I enjoy a challenge |
|  | _19_ | Able to work autonomously |
|  | _20_ | I am not afraid of confrontation |
|  | _21_ | I like sharing what I have learnt |
|  | _22_ | I have an intuitive approach to nursing/midwifery |
|  | _23_ | Solutions focused |
|  | _24_ | Other – please describe |
|  |  |  |

| **2.07** | **Of the statements that you have ticked from question 2.06, which is the MOST important reason that assisted you to achieve your current level of 'expertise'? (please choose one reason from question 2.06 and write down the number)** |
| --- | --- |
|  |  |

| **2.08** | **Thinking about your future in nursing or midwifery, on a scale of ‘1’ to ‘7’, where ‘1’ is ‘beginner’ and ‘7’ is ‘expert’ practitioner; please rate the level of ‘expertise’ that you would like to reach?**  **(If you feel that you have reached the level of ‘expertise’ that you want to be in nursing or midwifery, indicate the same number you did in the previous scale)** | | | | | | |
| --- | --- | --- | --- | --- | --- | --- | --- |
|  | **Beginner** |  |  |  |  |  | **Expert** |
|  | 1 | 2 | 3 | 4 | 5 | 6 | 7 |

| **2.09** | **Are there any factors preventing you from developing this higher level of ‘expertise’?** **(tick all that apply)** | |
| --- | --- | --- |
|  | **_1_** | I am practicing at my optimal level of expertise |
|  | **_2_** | I am comfortable practising at my current level |
|  | **_3_** | Not enough support from my manager |
|  | **_4_** | Not enough support from my colleagues |
|  | **_5_** | It’s difficult to adapt to change |
|  | **_6_** | I lack confidence in myself |
|  | **_7_** | I lack confidence in my abilities |
|  | **_8_** | Not enough role-specific knowledge |
|  | **_9_** | Too busy to find the time needed for development |
|  | **_10_** | Too early in my career |
|  | **_11_** | I have not been exposed to many situations |
|  | **_12_** | It costs too much money to further my development |
|  | **_13_** | Working part time is a barrier |
|  | **_14_** | I have had a leave of absence |
|  | **_15_** | I haven’t had enough opportunities |
|  | **_16_** | I am ‘put off’ by taking on more responsibilities from what my colleagues go through |
|  | **_17_** | I am ‘burnt out’ |
|  | **_18_** | Constant changes in the roster (changes in skill mix, being moved from rostered area) make it difficult |
|  | **_19_** | Working in one area makes it difficult |
|  | **_20_** | Working in multiple areas makes it difficult |
|  | **_21_** | Limited roles available to progress further |
|  | **_22_** | Work is a method of providing income only |
|  | **_23_** | I need a financial inducement |
|  | **_24_** | There is a culture of blame that prevents me from developing ‘expertise’ |
|  | **_25_** | The culture at the **[organisation]** prevents me from moving forward |
|  | **_26_** | I haven’t found the ‘right’ role for me yet, that I want to be an ‘expert’ in |
|  | **_27_** | Other – Please describe |
|  |  |  |

| **2.10** | **Of the statements that you ticked from question 2.09, which would you say is the MOST important reason that you have yet to develop further ‘expertise’? (please choose one factor from question 2.09 and write down the number)** |
| --- | --- |
|  |  |

|  |  | | | |
| --- | --- | --- | --- | --- |
| **2.11** | **Are there things that you need to further develop your ‘expertise’? (tick all that apply)** | | | |
|  | **_1_** | I don’t need anything | | |
|  | **_2_** | Reimbursement of costs for professional development | | |
|  | **_3_** | Further studies i.e. cert/masters/PHD | | |
|  | **_4_** | Paid professional development days | | |
|  | **_5_** | More protected learning time | | |
|  | **_6_** | Financial incentives | | |
|  | **_7_** | A mentorship/preceptorship program | | |
|  | **_8_** | Attending a leadership program | | |
|  | **_9_** | More opportunities to develop advanced skills | | |
|  | **_10_** | Opportunities to attend conferences | | |
|  | **_11_** | Leadership workshops | | |
|  | **_12_** | Education opportunities on night duty | | |
|  | **_13_** | Education opportunities on weekends | | |
|  | **_14_** | Access to clinical support staff who work night duty | | |
|  | **_15_** | Access to clinical support staff who work weekends | | |
|  | **_16_** | Supernumerary time in other roles | | |
|  | **_17_** | Supernumerary time to develop advanced skills | | |
|  | **_18_** | Support and encouragement from my manager | | |
|  | **_19_** | Support from my colleagues | | |
|  | **_20_** | Opportunities to assist in clinical practice changes | | |
|  | **_21_** | Positive feedback | | |
|  | **_22_** | Other – please describe | | |
|  |  | | | |
| **2.12** | **Of these ticked from question 2.11 which is the MOST important? (please choose one factor from question 2.11 and write down the number)** | | | |
|  |  | | | |
|  | **If your position is a clinical support nurse or midwife or educator, please go to question 2.17** | | | |
| **2.13** | **Would you consider applying for a clinical support nurse or midwife or clinical educator position in the next 12 months?**  (The clinical support role facilitates through the provision of direct clinical support, clinical learning, guiding, supporting and evaluating all nurses and midwives at the **[organisation]** including but not limited to new graduates, new staff and students. The clinical educator role is a nurse or midwife, employed to teach the theory and practice of nursing/midwifery) | | | |
|  | **_1_** | Yes | | |
|  | **_2_** | No *(go to question 2.16)* | | |
| **2.14** | **What do you need to achieve this position? (tick all that apply)** | | | |
|  | **_1_** | | Nothing, I feel I am already qualified for this position | |
|  | **_2_** | | Further knowledge of the role | |
|  | **_3_** | | Role specific skills | |
|  | **_4_** | | A position to become available | |
|  | **_5_** | | Motivation to apply | |
|  | **_6_** | | Further qualifications | |
|  | **_7_** | | Clinical experience | |
|  | **_8_** | | Confidence in myself to perform in the role | |
|  | **_9_** | | Other - please describe: | |
|  |  | | | |
| **2.15** | **What are the barriers to you achieving this position? (tick all that apply)** | | | |
|  | **_1_** | | None, I just haven’t applied yet | |
|  | **_2_** | | I need to work less than 0.6 EFT | |
|  | **_3_** | | Need more knowledge to achieve this role | |
|  | **_4_** | | Need more clinical skills to achieve this role | |
|  | **_5_** | | There are no positions available | |
|  | **_6_** | | I am ‘put off’ by what others experience in this position | |
|  | **_7_** | | Other - please describe: | |
|  |  | | | |
|  | **Please now go to question 2.17** | | | |
| **2.16** | **What are your reasons for not considering this position? (tick all that apply)** | | | |
|  | **_1_** | | I enjoy my current position, do not want to change | |
|  | **_2_** | | This position would be too stressful | |
|  | **_3_** | | My focus in my career is going in a different direction | |
|  | **_4_** | | I don’t like working with students or junior staff | |
|  | **_5_** | | I don’t think I would enjoy this role | |
|  | **_6_** | | I have worked this position before and am not interested at this time | |
|  | **_7_** | | Other - please describe: | |
|  |  | | | |
|  | **If your position is AUM please go to question 2.21** | | | |
| **2.17** | **Would you consider applying for an associate unit manager (AUM) position within the next 12 months?**  (AUM assumes the responsibilities and authority of the Team leader/Unit manager in their absence and as such supports the Team leader/Unit manager to provide day to day leadership and management of the team) | | | |
|  | **_1_** | Yes | | |
|  | **_2_** | No *(go to question 2.20)* | | |
| **2.18** | **What do you need to achieve this position? (tick all that apply)** | | | |
|  | **_1_** | | Nothing, I feel I am already qualified for this position | |
|  | **_2_** | | Further knowledge of the role | |
|  | **_3_** | | Role-specific skills | |
|  | **_4_** | | A position to become available | |
|  | **_5_** | | Motivation to apply | |
|  | **_6_** | | Clinical experience | |
|  | **_7_** | | Confidence in myself to perform in the role | |
|  | **_8_** | | Manager support | |
|  | **_9_** | | Leadership program | |
|  | **_10_** | | Other - please describe: | |
|  |  | | | |
| **2.19** | **What are the barriers to you achieving this position? (tick all that apply)** | | | |
|  | **_1_** | | None, I just haven’t applied yet | |
|  | **_2_** | | I work part time – no part time positions available | |
|  | **_3_** | | Need more knowledge to achieve this role | |
|  | **_4_** | | Need more clinical skills to achieve this role | |
|  | **_5_** | | There are no positions available | |
|  | **_6_** | | My manager would not support me in this | |
|  | **_7_** | | I want to work in one area, this position would require me to work in multiple areas | |
|  | **_8_** | | I am ‘put off’ by what other people experience in this position | |
|  | **_9_** | | Other - please describe: | |
|  |  | | | |
|  | **Please now go to question 2.21** | | | |
| **2.20** | **What are your reasons for not considering this position? (tick all that apply)** | | | |
|  | **_1_** | | I enjoy my current position, and do not want to change | |
|  | **_2_** | | This position would be too stressful | |
|  | **_3_** | | My focus in my career is going in a different direction | |
|  | **_4_** | | Prefer hands-on patient care | |
|  | **_5_** | | Don’t want to deal with the issues of skill mix | |
|  | **_6_** | | I don’t want to work shift work | |
|  | **_7_** | | My manager wouldn’t support me to achieve this position | |
|  | **_8_** | | I don’t think I would enjoy this role | |
|  | **_9_** | | This would take away from my work/life balance | |
|  | **_10_** | | My family responsibilities would make this role too difficult | |
|  | **_11_** | | I have done this position before and am not interested at this time | |
|  | **_12_** | | The financial incentive does not match the level of responsibility | |
|  | **_13_** | | Other - please describe: | |
|  |  | | | |
|  | **If you are a nurse practitioner, please go to question 2.25** | | | |
| **2.21** | **Would you undertake further study to become a nurse practitioner in the next 1-5 years?**  (Currently the most advanced clinical nursing role, nurse practitioner’s scope of practice is determined by the context in which each nurse practitioner is authorised to practice and may involve referral of patients to other health care professionals, prescribing medicines and ordering diagnostic investigations. It requires minimum 5 years of nursing experience, 3 years in a specialised field and 1 of those years at an advanced practice level. A program of study i.e. master’s also needs to be completed) | | | |
|  | **_1_** | Yes | | |
|  | **_2_** | No *(go to question 2.24)* | | |
| **2.22** | **What do you need to undertake study to become a nurse practitioner? (tick all that apply)** | | | |
|  | **_1_** | | I am already studying for this | |
|  | **_2_** | | Further knowledge of the role | |
|  | **_3_** | | Role-specific skills | |
|  | **_4_** | | A position to become available | |
|  | **_5_** | | Motivation to apply | |
|  | **_6_** | | Further qualifications | |
|  | **_7_** | | Clinical experience | |
|  | **_8_** | | Manager support | |
|  | **_9_** | | Other - please describe: | |
|  |  | | | |
| **2.23** | **What are the barriers to you undertaking study to become a nurse practitioner? (tick all that apply)** | | | |
|  | **_1_** | | None, I just haven’t applied yet | |
|  | **_2_** | | I work full time | |
|  | **_3_** | | I need a mentor to show me the way | |
|  | **_4_** | | Need more knowledge to study | |
|  | **_5_** | | There are no positions available | |
|  | **_6_** | | My manager would not support me in this | |
|  | **_7_** | | I don’t really understand what the role involves | |
|  | **_8_** | | No time | |
|  | **_9_** | | Cannot afford it at this stage | |
|  | **_10_** | | Other - please describe: | |
|  |  | | | |
|  | **Please now go to question 2.25** | | | |
| **2.24** | **What are the reasons that you would not consider studying to become a nurse practitioner? (tick all that apply)** | | | |
|  | **_1_** | | I enjoy my current position, do not want to change | |
|  | **_2_** | | I work full time | |
|  | **_3_** | | I don’t really understand what the role involves | |
|  | **_4_** | | This study is not applicable to my midwifery role | |
|  | **_5_** | | My focus in my career is going in a different direction | |
|  | **_6_** | | I am not interested in any positions that involve research | |
|  | **_7_** | | My manager wouldn’t support me to achieve this position | |
|  | **_8_** | | I don’t think I would enjoy this role | |
|  | **_9_** | | No time | |
|  | **_10_** | | This role isn’t applicable to my practice | |
|  | **_11_** | | There are no benefits for me in this role | |
|  | **_12_** | | My family responsibilities prevent me from doing this at this stage | |
|  | **_13_** | | Other - please describe: | |
|  |  | | | |
|  | **If you currently are an eligible midwife, please go to question 2.29** | | | |
| **2.25** | **Would you undergo further training to become an eligible midwife in the next 1-5 years?**  (Having notation as an eligible midwife on the register of midwives indicates that the midwife is competent to: provide pregnancy, labour, birth and postnatal care to women and their infants; and is qualified to provide the associated services and order diagnostic investigations appropriate to the eligible midwife’s scope of practice. It requires a minimum of 3 years full time experience, completion of a professional practice review program and applicable program of study and an extra 20 hours of CPD to qualify) | | | |
|  | **_1_** | Yes | | |
|  | **_2_** | No *(go to question 2.28)* | | |
| **2.26** | **What do you need to undertake further training to become an eligible midwife? (tick all that apply)** | | | |
|  | **_1_** | | I am already studying for this | |
|  | **_2_** | | Further knowledge of the role | |
|  | **_3_** | | Time to complete the course work/practice review | |
|  | **_4_** | | Motivation to apply | |
|  | **_5_** | | More years of experience | |
|  | **_6_** | | Manager support | |
|  | **_7_** | | Other - please describe: | |
|  |  | | | |
| **2.27** | **What are the barriers to you undertaking further training to become an eligible midwife? (tick all that apply)** | | | |
|  | **_1_** | | None, I just haven’t applied yet | |
|  | **_2_** | | I work full time | |
|  | **_3_** | | I need a mentor to show me the way | |
|  | **_4_** | | Need more knowledge to study | |
|  | **_5_** | | It currently doesn’t apply to my practice | |
|  | **_6_** | | My manager would not support me in this | |
|  | **_7_** | | I don’t really understand what the role involves | |
|  | **_8_** | | No time | |
|  | **_9_** | | Cannot afford it at this stage | |
|  | **_10_** | | Other - please describe: | |
|  |  | |  | |
|  |  | | **Please now go to question 2.29** | |
| **2.28** | **What are the reasons that you would not consider further training to become an eligible midwife? (tick all that apply)** | | | |
|  | **_1_** | | I enjoy my current position, do not want to change | |
|  | **_2_** | | I work full time | |
|  | **_3_** | | I don’t really understand what the role involves | |
|  | **_4_** | | This study is not applicable to my nursing role | |
|  | **_5_** | | My focus in my career is going in a different direction | |
|  | **_6_** | | This further notation would be of no benefit to my current position | |
|  | **_7_** | | No time | |
|  | **_8_** | | This role isn’t applicable to my practice | |
|  | **_9_** | | There are no benefits for me in this role | |
|  | **_10_** | | My family responsibilities prevent me from doing this at this stage | |
|  | **_11_** | | Other - please describe: | |
|  | **If your position is a clinical nurse or midwife consultant, please go to question 2.33** | | | |
| **2.29** | **Would you consider applying for a clinical nurse or midwife consultant position in the next 1-5 years?**  (The CNC/CMC is varied but may include providing expert clinical advice to patients, carers and other health care professionals within a defined specialty. The CNC/CMC usually participates in formal processes for the strategic and operational planning for the clinical service. The CNC/CMC provides leadership that facilitates the ongoing development of clinical practice. The role may also involve the organisation and delivery of specialist consultant services. The CNC/CMC role also usually contributes to the development and delivery of specialty related education programs and initiates and utilises findings of research in the provision of clinical services.) | | | |
|  | **_1_** | Yes | | |
|  | **_2_** | No *(go to question 2.32)* | | |
| **2.30** | **What do you need to achieve this position? (tick all that apply)** | | | |
|  | **_1_** | | Nothing, I am already qualified for this position | |
|  | **_2_** | | Further knowledge of the role | |
|  | **_3_** | | Role-specific skills | |
|  | **_4_** | | A position to become available | |
|  | **_5_** | | Motivation to apply | |
|  | **_6_** | | Further qualifications | |
|  | **_7_** | | Clinical experience | |
|  | **_8_** | | Confidence in myself to perform in the role | |
|  | **_9_** | | Manager support | |
|  | **_10_** | | Other - please describe: | |
|  |  | | | |
| **2.31** | **What are the barriers to you achieving this position? (tick all that apply)** | | | |
|  | **_1_** | | None, I just haven’t applied yet | |
|  | **_2_** | | I work part time – no part time positions available | |
|  | **_3_** | | I need a mentor to show me the way | |
|  | **_4_** | | Need more knowledge to achieve this role | |
|  | **_5_** | | Need more clinical skills to achieve this role | |
|  | **_6_** | | There are no positions available | |
|  | **_7_** | | My manager would not support me in this | |
|  | **_8_** | | I don’t really understand what the role involves | |
|  | **_9_** | | No time to achieve this position | |
|  | **_10_** | | I want to work in multiple areas, this position confines me to one area | |
|  | **_11_** | | Other - please describe: | |
|  |  | | | |
|  | **Please now go to question 2.33** | | | |
| **2.32** | **What are your reasons for not considering this position (tick all that apply)** | | | |
|  | **_1_** | | I enjoy my current position, do not want to change | |
|  | **_2_** | | This position would be too stressful | |
|  | **_3_** | | My focus in my career is going in a different direction | |
|  | **_4_** | | I am ‘put off’ by what other people experience in this position | |
|  | **_5_** | | I am not interested in any positions that involve research | |
|  | **_6_** | | My manager wouldn’t support me to achieve this position | |
|  | **_7_** | | I don’t think I would enjoy this role | |
|  | **_8_** | | This would take away from my work/life balance | |
|  | **_9_** | | I have done this position before | |
|  | **_10_** | | The financial incentive does not match the level of responsibility | |
|  | **_11_** | | Other - please describe: | |
|  |  | | | |
|  | **All participants please answer this question** | | | |
| **2.33** | **Would you consider applying for a management position or higher management position than the one you currently hold in the next 1-5 years?** | | | |
|  | **_1_** | Yes | | |
|  | **_2_** | No *(go to question 2.37)* | | |
| **2.34** | **Which positions would you apply for if they were available? (Tick all that apply)** | | | |
|  | **_1_** | | | Team leader/Unit manager |
|  | **_2_** | | | Director |
|  | **_3_** | | | Executive Director |
|  | **_4_** | | | Other - please describe: |
| **2.35** | **What do you need to achieve these positions? (Tick all that apply)** | | | |
|  | **_1_** | | Nothing, I am already qualified for this position | |
|  | **_2_** | | Further knowledge of the role | |
|  | **_3_** | | Role-specific skills | |
|  | **_4_** | | A position to become available | |
|  | **_5_** | | Motivation to apply | |
|  | **_6_** | | Further qualifications | |
|  | **_7_** | | Confidence in myself to perform in the role | |
|  | **_8_** | | Manager support | |
|  | **_9_** | | Other - please describe: | |
|  |  | | | |
| **2.36** | **What are the barriers to you achieving these positions? (tick all that apply)** | | | |
|  | **_1_** | | None, I just haven’t applied yet | |
|  | **_2_** | | I have no management experience | |
|  | **_3_** | | I work part time – no part time positions available | |
|  | **_4_** | | I need a mentor to show me the way | |
|  | **_5_** | | Need more knowledge to achieve this role | |
|  | **_6_** | | There are no positions available | |
|  | **_7_** | | My manager would not support me in this | |
|  | **_8_** | | I don’t really understand what the role involves | |
|  | **_9_** | | I am worried that I could not do the role | |
|  | **_10_** | | I want to work in multiple areas, this position confines me to one area | |
|  | **_11_** | | I am ‘put off’ by what other people experience in this position | |
|  | **_12_** | | Other - please describe: | |
|  |  | | | |
|  | **Please now go to question 2.38** | | | |
| **2.37** | **What are your reasons for not considering these positions? (tick all that apply)** | | | |
|  | **_1_** | I enjoy my current position, do not want to change | | |
|  | **_2_** | This position would be too stressful | | |
|  | **_3_** | My focus in my career is going in a different direction | | |
|  | **_4_** | I don’t want to stop working in a clinical role | | |
|  | **_5_** | Don’t want to deal with the issues of skill mix | | |
|  | **_6_** | My manager wouldn’t support me to achieve this position | | |
|  | **_7_** | I don’t have any budget/business experience | | |
|  | **_8_** | I don’t think I would enjoy this role | | |
|  | **_9_** | I have no interest in management positions | | |
|  | **_10_** | This would take away from my work/life balance | | |
|  | **_11_** | I have done this position before and am not interested at this time | | |
|  | **_12_** | The financial incentive does not match the level of responsibility | | |
|  | **_13_** | My family responsibilities would make this role too difficult | | |
|  | **_14_** | Other - please describe: | | |
|  |  | | | |
| **2.38** | **What do you think prevents or deters other nurses or midwives from becoming ‘experts’ in their field? (tick all that apply)** | | | |
|  | **_1_** | Heavy workload | | |
|  | **_2_** | Lack of interest | | |
|  | **_3_** | They consider themselves ‘experts’ already | | |
|  | **_4_** | Not enough exposure to complex situations | | |
|  | **_5_** | Lack of peer support | | |
|  | **_6_** | Lack of manager support | | |
|  | **_7_** | Afraid to ask questions | | |
|  | **_8_** | Unwilling to learn | | |
|  | **_9_** | Lack of finances to continue further education | | |
|  | **_10_** | They are burnt out | | |
|  | **_11_** | Can’t adapt to change or new situations | | |
|  | **_12_** | They don’t have a mentor | | |
|  | **_13_** | They are ‘put off’ by taking on more responsibilities from what their colleagues go through | | |
|  | **_14_** | No confidence in themselves | | |
|  | **_15_** | Poor communicators | | |
|  | **_16_** | They don’t engage well with staff/patients | | |
|  | **_17_** | There is a culture of blame that prevents them from becoming ‘experts’ | | |
|  | **_18_** | The culture at the **[organisation]** prevents them from moving forward | | |
|  | **_19_** | Other - please describe: | | |
|  |  | | | |
| **2.39** | **Of the statements that you have ticked from question 2.38, which is the MOST important reason that prevents or deters other nurses or midwives from becoming 'experts' in their field? (please choose one from question 2.38 and write down the number)** | | | |
|  |  | | | |

| **Section 3: What it’s like for you to work at the [organisation]** | | | | | | | | | | | | | | | | | | | | | | | | | | | | | | | | | | | | | | |  |
| --- | --- | --- | --- | --- | --- | --- | --- | --- | --- | --- | --- | --- | --- | --- | --- | --- | --- | --- | --- | --- | --- | --- | --- | --- | --- | --- | --- | --- | --- | --- | --- | --- | --- | --- | --- | --- | --- | --- | --- |
| **3.01** | **What are the key features for an ‘enjoyable’ shift or day for you**? (tick all that apply) | | | | | | | | | | | | | | | | | | | | | | | | | | | | | | | | | | | | | |  |
|  | **_1_** | | | Working with colleagues that have the same values as me | | | | | | | | | | | | | | | | | | | | | | | | | | | | | | | | | | |  |
|  | **_2_** | | | Knowing the majority of the staff on the shift/day | | | | | | | | | | | | | | | | | | | | | | | | | | | | | | | | | | |  |
|  | **_3_** | | | Colleagues with a good work ethic | | | | | | | | | | | | | | | | | | | | | | | | | | | | | | | | | | |  |
|  | **_4_** | | | Having mostly senior staff on | | | | | | | | | | | | | | | | | | | | | | | | | | | | | | | | | | |  |
|  | **_5_** | | | Being busy | | | | | | | | | | | | | | | | | | | | | | | | | | | | | | | | | | |  |
|  | **_6_** | | | Having a ‘quiet’ shift/day | | | | | | | | | | | | | | | | | | | | | | | | | | | | | | | | | | |  |
|  | **_7_** | | | No staff from the management team present | | | | | | | | | | | | | | | | | | | | | | | | | | | | | | | | | | |  |
|  | **_8_** | | | Having a ‘good’ in charge nurse or midwife | | | | | | | | | | | | | | | | | | | | | | | | | | | | | | | | | | |  |
|  | **_9_** | | | Getting all my work done | | | | | | | | | | | | | | | | | | | | | | | | | | | | | | | | | | |  |
|  | **_10_** | | | Being able to provide high level of care to women and families | | | | | | | | | | | | | | | | | | | | | | | | | | | | | | | | | | |  |
|  | **_11_** | | | Fewer patients in the ward than the current norm | | | | | | | | | | | | | | | | | | | | | | | | | | | | | | | | | | |  |
|  | **_12_** | | | Working with my ‘friends” | | | | | | | | | | | | | | | | | | | | | | | | | | | | | | | | | | |  |
|  | **_13_** | | | Being able to provide care to women and families in a way that reflects my underlying philosophy of being a nurse or midwife | | | | | | | | | | | | | | | | | | | | | | | | | | | | | | | | | | |  |
|  | **_14_** | | | Appropriate staff skill mix | | | | | | | | | | | | | | | | | | | | | | | | | | | | | | | | | | |  |
|  | **_15_** | | | Other - please describe: | | | | | | | | | | | | | | | | | | | | | | | | | | | | | | | | | | |  |
|  |  | | | | | | | | | | | | | | | | | | | | | | | | | | | | | | | | | | | | | |  |
| **3.02** | **Of these ticked from question 3.01, which is the MOST important? (please choose one answer from question 3.01 and write down the number)** | | | | | | | | | | | | | | | | | | | | | | | | | | | | | | | | | | | | | |  |
|  |  | | | | | | | | | | | | | | | | | | | | | | | | | | | | | | | | | | | | | |  |
| **3.03** | **What characteristics do you rate highly in a co-worker?** (**Tick all that apply)** | | | | | | | | | | | | | | | | | | | | | | | | | | | | | | | | | | | | | |  |
|  | **_1_** | | | Approachability | | | | | | | | | | | | | | | | | | | | | | | | | | | | | | | | | | |  |
|  | **_2_** | | | Knowledge | | | | | | | | | | | | | | | | | | | | | | | | | | | | | | | | | | |  |
|  | **_3_** | | | Leadership skills | | | | | | | | | | | | | | | | | | | | | | | | | | | | | | | | | | |  |
|  | **_4_** | | | Passion | | | | | | | | | | | | | | | | | | | | | | | | | | | | | | | | | | |  |
|  | **_5_** | | | Humour | | | | | | | | | | | | | | | | | | | | | | | | | | | | | | | | | | |  |
|  | **_6_** | | | Understanding | | | | | | | | | | | | | | | | | | | | | | | | | | | | | | | | | | |  |
|  | **_7_** | | | Confidence | | | | | | | | | | | | | | | | | | | | | | | | | | | | | | | | | | |  |
|  | **_8_** | | | Team player | | | | | | | | | | | | | | | | | | | | | | | | | | | | | | | | | | |  |
|  | **_9_** | | | Positivity | | | | | | | | | | | | | | | | | | | | | | | | | | | | | | | | | | |  |
|  | **_10_** | | | Years of experience – if so, how many years? | | | | | | | | | | | | | | | | | | | | | | | | | | | | | | | | | | |  |
|  |  | | | | | **_1_** | ≤3 years | | | | | | | | | | | | | | | | | | | | | | | | | | | | | | | |  |
|  |  | | | | | **_2_** | 3-5 years | | | | | | | | | | | | | | | | | | | | | | | | | | | | | | | |  |
|  |  | | | | | **_3_** | 6-10 years | | | | | | | | | | | | | | | | | | | | | | | | | | | | | | | |  |
|  |  | | | | | **_4_** | >10 years | | | | | | | | | | | | | | | | | | | | | | | | | | | | | | | |  |
|  | **_11_** | | | Other - please describe: | | | | | | | | | | | | | | | | | | | | | | | | | | | | | | | | | | |  |
|  |  | | | | | | | | | | | | | | | | | | | | | | | | | | | | | | | | | | | | | |  |
| **3.04** | **Of these ticked from question 3.03, which is the MOST important? (please choose one from question 3.03 and write down the number)** | | | | | | | | | | | | | | | | | | | | | | | | | | | | | | | | | | | | | |  |
|  |  | | | | | | | | | | | | | | | | | | | | | | | | | | | | | | | | | | | | | |  |
| **3.05** | **How often would you see the following behaviours amongst staff (any staff) at the [organisation]?** | | | | | | | | | | | | | | | | | | | | | | | | | | | | | | | | | | | | | |  |
|  |  | | | | | | | | | | | | **Never** | | | | | | **Rarely** | | | | | | **Sometimes** | | | | | **Frequently** | | | | | | **Always** | | |  |
|  | a) Favouritism to certain staff members | | | | | | | | | | | | 1 | | | | | | 2 | | | | | | 3 | | | | | 4 | | | | | | 5 | | |  |
|  | b) Refusal to help or support others | | | | | | | | | | | | 1 | | | | | | 2 | | | | | | 3 | | | | | 4 | | | | | | 5 | | |  |
|  | c) Negative feedback given unconstructively | | | | | | | | | | | | 1 | | | | | | 2 | | | | | | 3 | | | | | 4 | | | | | | 5 | | |  |
|  | d) Threatening or abusive language | | | | | | | | | | | | 1 | | | | | | 2 | | | | | | 3 | | | | | 4 | | | | | | 5 | | |  |
|  | e) Humiliation of someone in front of others, including staff and/or patients | | | | | | | | | | | | 1 | | | | | | 2 | | | | | | 3 | | | | | 4 | | | | | | 5 | | |  |
|  |  | | | | | | | | | | | | **Never** | | | | | | **Rarely** | | | | | | **Sometimes** | | | | | **Frequently** | | | | | | **Always** | | |  |
|  | f) Reluctance or refusal to answer questions | | | | | | | | | | | | 1 | | | | | | 2 | | | | | | 3 | | | | | 4 | | | | | | 5 | | |  |
|  | g) Throwing items | | | | | | | | | | | | 1 | | | | | | 2 | | | | | | 3 | | | | | 4 | | | | | | 5 | | |  |
|  | h) Self-absorbed about their own problems | | | | | | | | | | | | 1 | | | | | | 2 | | | | | | 3 | | | | | 4 | | | | | | 5 | | |  |
|  | i) ‘Whining’ and complaining | | | | | | | | | | | | 1 | | | | | | 2 | | | | | | 3 | | | | | 4 | | | | | | 5 | | |  |
|  | j) Unreasonable demands made of you or others | | | | | | | | | | | | 1 | | | | | | 2 | | | | | | 3 | | | | | 4 | | | | | | 5 | | |  |
|  | k) Talking negatively about women and families | | | | | | | | | | | | 1 | | | | | | 2 | | | | | | 3 | | | | | 4 | | | | | | 5 | | |  |
|  | l) Talking negatively about colleagues or co-workers | | | | | | | | | | | | 1 | | | | | | 2 | | | | | | 3 | | | | | 4 | | | | | | 5 | | |  |
|  | m) Refusing to mentor students or junior staff | | | | | | | | | | | | 1 | | | | | | 2 | | | | | | 3 | | | | | 4 | | | | | | 5 | | |  |
|  | n) Devaluing your skills or expertise | | | | | | | | | | | | 1 | | | | | | 2 | | | | | | 3 | | | | | 4 | | | | | | 5 | | |  |
| **3.06** | **When talking to nurses and midwives about the staff skill mix on a shift, occasionally it has been described as ‘unsafe’. What does ‘unsafe’ mean to you when referring to skill mix?** | | | | | | | | | | | | | | | | | | | | | | | | | | | | | | | | | | | | | |  |
|  |  | | | | | | | | | | | | | | | | | | | | | | | | | | | | | | | | | | | | | |  |
| **3.07** | **Currently; in an average week at the [organisation] in your opinion how often is the staff skill mix on each shift ‘unsafe’?** | | | | | | | | | | | | | | | | | | | | | | | | | | | | | | | | | | | | | |  |
|  | **_1_** | | | None of the time | | | | | | | | | | | | | | | | | | | | | | | | | | | | | | | | | | |  |
|  | **_2_** | | | Little of the time | | | | | | | | | | | | | | | | | | | | | | | | | | | | | | | | | | |  |
|  | **_3_** | | | Some of the time | | | | | | | | | | | | | | | | | | | | | | | | | | | | | | | | | | |  |
|  | **_4_** | | | Most of the time | | | | | | | | | | | | | | | | | | | | | | | | | | | | | | | | | | |  |
|  | **_5_** | | | All of the time | | | | | | | | | | | | | | | | | | | | | | | | | | | | | | | | | | |  |
| **3.08** | **Other than employing more staff, what could be done to improve skill mix on a day-by-day basis?** | | | | | | | | | | | | | | | | | | | | | | | | | | | | | | | | | | | | | |  |
|  |  | | | | | | | | | | | | | | | | | | | | | | | | | | | | | | | | | | | | | |  |
| **3.09** | **The next statements involve your views on your decision making and support within nursing and midwifery (please tick one number for each statement)** | | | | | | | | | | | | | | | | | | | | | | | | | | | | | | | | | | | | | |  |
|  |  | | | | | | | | **Never** | | | | | | **Rarely** | | | | | **Sometimes** | | | | | | **Often** | | | | | | **Almost always** | | | | | **NA** | |  |
|  | a) I am confident in identifying clinical deterioration | | | | | | | | 1 | | | | | | 2 | | | | | 3 | | | | | | 4 | | | | | | 5 | | | | | 6 | |  |
|  | b) I am worried about approaching senior staff for assistance | | | | | | | | 1 | | | | | | 2 | | | | | 3 | | | | | | 4 | | | | | | 5 | | | | | 6 | |  |
|  | c) I feel like my clinical decisions are supported by my senior staff even if they conflict with medical decisions | | | | | | | | 1 | | | | | | 2 | | | | | 3 | | | | | | 4 | | | | | | 5 | | | | | 6 | |  |
|  | d) My concerns when raised with senior staff are NOT taken seriously | | | | | | | | 1 | | | | | | 2 | | | | | 3 | | | | | | 4 | | | | | | 5 | | | | | 6 | |  |
|  | e) There have been occasions where I haven’t identified a deterioration in the patient’s condition | | | | | | | | 1 | | | | | | 2 | | | | | 3 | | | | | | 4 | | | | | | 5 | | | | | 6 | |  |
|  | f) I am confident in approaching senior staff with my concerns | | | | | | | | 1 | | | | | | 2 | | | | | 3 | | | | | | 4 | | | | | | 5 | | | | | 6 | |  |
| **3.10** | **In the last month I have asked a staff member with equal or less experience for clinical advice:** | | | | | | | | | | | | | | | | | | | | | | | | | | | | | | | | | | | | | |  |
|  | **_1_** | | Never *(go to question 3.12)* | | | | | | | | | | | | | | | | | | | | | | | | | | | | | | | | | | | |  |
|  | **_2_** | | Once | | | | | | | | | | | | | | | | | | | | | | | | | | | | | | | | | | | |  |
|  | **_3_** | | 2-3 times | | | | | | | | | | | | | | | | | | | | | | | | | | | | | | | | | | | |  |
|  | **_4_** | | 4-5 times | | | | | | | | | | | | | | | | | | | | | | | | | | | | | | | | | | | |  |
|  | **_5_** | | >5 times | | | | | | | | | | | | | | | | | | | | | | | | | | | | | | | | | | | |  |
| **3.11** | **Why did you choose to ask a staff member with equal or less experience for advice? (tick all that apply)** | | | | | | | | | | | | | | | | | | | | | | | | | | | | | | | | | | | | | |  |
|  | **_1_** | | No senior staff member available | | | | | | | | | | | | | | | | | | | | | | | | | | | | | | | | | | | |  |
|  | **_2_** | | I was the most senior staff member at the time | | | | | | | | | | | | | | | | | | | | | | | | | | | | | | | | | | | |  |
|  | **_3_** | | They work in the area more often than I do | | | | | | | | | | | | | | | | | | | | | | | | | | | | | | | | | | | |  |
|  | **_4_** | | They have worked in the area more recently than I have | | | | | | | | | | | | | | | | | | | | | | | | | | | | | | | | | | | |  |
|  | **_5_** | | The senior staff available were too intimidating to ask | | | | | | | | | | | | | | | | | | | | | | | | | | | | | | | | | | | |  |
|  | **_6_** | | I wanted a second opinion on my decision | | | | | | | | | | | | | | | | | | | | | | | | | | | | | | | | | | | |  |
|  | **_7_** | | I wanted to discuss my problem ‘out loud’ | | | | | | | | | | | | | | | | | | | | | | | | | | | | | | | | | | | |  |
|  | **_8_** | | Other - please describe: | | | | | | | | | | | | | | | | | | | | | | | | | | | | | | | | | | | |  |
|  |  | |  | | | | | | | | | | | | | | | | | | | | | | | | | | | | | | | | | | | |  |
| **3.12** | **Do you feel that you need more support in order to fulfil your current role?** | | | | | | | | | | | | | | | | | | | | | | | | | | | | | | | | | | | | | |  |
|  | **_1_** | | Yes | | | | | | | | | | | | | | | | | | | | | | | | | | | | | | | | | | | |  |
|  | **_2_** | | No *(go to question 3.14)* | | | | | | | | | | | | | | | | | | | | | | | | | | | | | | | | | | | |  |
| **3.13** | **What would this support look like to you? (tick all that apply)** | | | | | | | | | | | | | | | | | | | | | | | | | | | | | | | | | | | | | |  |
|  | **_1_** | | | My manager having more input in my role | | | | | | | | | | | | | | | | | | | | | | | | | | | | | | | | | | |  |
|  | **_2_** | | | More access to clinical support staff | | | | | | | | | | | | | | | | | | | | | | | | | | | | | | | | | | |  |
|  | **_3_** | | | More time to attend professional development days | | | | | | | | | | | | | | | | | | | | | | | | | | | | | | | | | | |  |
|  | **_4_** | | | Feedback on how I can improve in my role | | | | | | | | | | | | | | | | | | | | | | | | | | | | | | | | | | |  |
|  | **_5_** | | | Feedback on what I am doing well within my role | | | | | | | | | | | | | | | | | | | | | | | | | | | | | | | | | | |  |
|  | **_6_** | | | Other - please describe: | | | | | | | | | | | | | | | | | | | | | | | | | | | | | | | | | | |  |
|  |  | | | | | | | | | | | | | | | | | | | | | | | | | | | | | | | | | | | | | |  |
| **3.14** | **Do you feel that you need more support in order to advance within your profession at the [organisation]?** | | | | | | | | | | | | | | | | | | | | | | | | | | | | | | | | | | | | | |  |
|  | **_1_** | Yes | | | | | | | | | | | | | | | | | | | | | | | | | | | | | | | | | | | | |  |
|  | **_2_** | No *(go to question 3.16)* | | | | | | | | | | | | | | | | | | | | | | | | | | | | | | | | | | | | |  |
| **3.15** | **What would this support look like to you? (tick all that apply)** | | | | | | | | | | | | | | | | | | | | | | | | | | | | | | | | | | | | | |  |
|  | **_1_** | | Supernumerary time in-charge roles | | | | | | | | | | | | | | | | | | | | | | | | | | | | | | | | | | | |  |
|  | **_2_** | | Supernumerary time in management roles | | | | | | | | | | | | | | | | | | | | | | | | | | | | | | | | | | | |  |
|  | **_3_** | | Leadership programs – e.g. AUM course | | | | | | | | | | | | | | | | | | | | | | | | | | | | | | | | | | | |  |
|  | **_4_** | | Supported further study – e.g. Masters | | | | | | | | | | | | | | | | | | | | | | | | | | | | | | | | | | | |  |
|  | **_5_** | | A mentor that is not affiliated with my role/area of work | | | | | | | | | | | | | | | | | | | | | | | | | | | | | | | | | | | |  |
|  | **_6_** | | A portfolio that I can be affiliated with | | | | | | | | | | | | | | | | | | | | | | | | | | | | | | | | | | | |  |
|  | **_7_** | | Secondment to other areas or roles to further my skills/knowledge | | | | | | | | | | | | | | | | | | | | | | | | | | | | | | | | | | | |  |
|  | **_8_** | | Other - please describe: | | | | | | | | | | | | | | | | | | | | | | | | | | | | | | | | | | | |  |
|  |  | | | | | | | | | | | | | | | | | | | | | | | | | | | | | | | | | | | | | |  |
| **3.16** | **In your opinion, is the role of clinical nurse or midwife specialist (CNS/CMS) utilised well at the [organisation]?**  (A CNS/CMS is defined as a registered nurse/midwife who applies a high level of clinical nursing knowledge, experience and skills in providing complex nursing/midwifery care directed towards a specific area of practice, a defined population or defined service area, with minimum direct supervision) | | | | | | | | | | | | | | | | | | | | | | | | | | | | | | | | | | | | | |  |
|  | **_1_** | Yes *(go to question 3.17)* | | | | | | | | | | | | | | | | | | | | | | | | | | | | | | | | | | | | |  |
|  | **_2_** | No *(go to question 3.18)* | | | | | | | | | | | | | | | | | | | | | | | | | | | | | | | | | | | | |  |
| **3.17** | **Please list your reasons as to why this position is well utilised** | | | | | | | | | | | | | | | | | | | | | | | | | | | | | | | | | | | | | |  |
| **3.18** | **Please list your reasons as to why this position isn't well utilised** | | | | | | | | | | | | | | | | | | | | | | | | | | | | | | | | | | | | | |  |
|  |  | | | | | | | | | | | | | | | | | | | | | | | | | | | | | | | | | | | | | |  |
| **3.19** | **In your opinion, is the role of associate unit manager (AUM) utilised well at the [organisation]?**  (AUM assumes the responsibilities and authority of the Team leader/Unit manager in their absence and as such supports the Team leader/Unit manager to provide day to day leadership and management of the team) | | | | | | | | | | | | | | | | | | | | | | | | | | | | | | | | | | | | | |  |
|  | **_1_** | | Yes *(go to question 3.20)* | | | | | | | | | | | | | | | | | | | | | | | | | | | | | | | | | | | |  |
|  | **_2_** | | No *(go to question 3.21)* | | | | | | | | | | | | | | | | | | | | | | | | | | | | | | | | | | | |  |
| **3.20** | **Please list your reasons as to why this position is well utilised** | | | | | | | | | | | | | | | | | | | | | | | | | | | | | | | | | | | | | |  |
| **3.21** | **Please list your reasons as to why this position isn't well utilised** | | | | | | | | | | | | | | | | | | | | | | | | | | | | | | | | | | | | | |  |
|  |  | | | | | | | | | | | | | | | | | | | | | | | | | | | | | | | | | | | | | |  |
| **3.22** | **Do you have clinical support nurses or midwives or clinical educators in your main area of work?** | | | | | | | | | | | | | | | | | | | | | | | | | | | | | | | | | | | | | |  |
|  | **_1_** | | Yes *(go to question 3.23)* | | | | | | | | | | | | | | | | | | | | | | | | | | | | | | | | | | | |  |
|  | **_2_** | | No *(go to question 3.25)* | | | | | | | | | | | | | | | | | | | | | | | | | | | | | | | | | | | |  |
| **3.23** | **In your opinion, are the roles of clinical support nurses or midwives or clinical educators utilised well at the [organisation]?**  (The clinical support role facilitates through the provision of direct clinical support, clinical learning, guiding, supporting and evaluating all nurses and midwives at the [organisation] including but not limited to new graduates, new staff and students. The clinical educator role is a nurse or midwife, employed to teach the theory and practice of nursing/midwifery) | | | | | | | | | | | | | | | | | | | | | | | | | | | | | | | | | | | | | |  |
|  | **_1_** | | Yes *(go to question 3.24)* | | | | | | | | | | | | | | | | | | | | | | | | | | | | | | | | | | | |  |
|  | **_2_** | | No *(go to question 3.25)* | | | | | | | | | | | | | | | | | | | | | | | | | | | | | | | | | | | |  |
| **3.24** | **Please list your reasons as to why this position is well utilised** | | | | | | | | | | | | | | | | | | | | | | | | | | | | | | | | | | | | | |  |
| **3.25** | **Please list your reasons as to why this position isn't well utilised** | | | | | | | | | | | | | | | | | | | | | | | | | | | | | | | | | | | | | |  |
|  |  | | | | | | | | | | | | | | | | | | | | | | | | | | | | | | | | | | | | | |  |
| **3.26** | **The following questions explore how you are feeling within work**  These questions relate to your current role. Where it states ‘women and their families’ this is related to your current role e.g. clinical midwife the women and their families would be the people you care for, for managers this would be the staff you manage.  Please check the most appropriate answer to each statement. | | | | | | | | | | | | | | | | | | | | | | | | | | | | | | | | | | | | | |  |
|  |  | | | | | | | | | | | **Never** | | | | | | **Seldom** | | | | | | **Sometimes** | | | | | **Often** | | | | | | **Always** | | | |  |
|  | a) Are you exhausted in the morning at the thought of another day at work? | | | | | | | | | | | **_1_** | | | | | | **_2_** | | | | | | **_3_** | | | | | **_4_** | | | | | | **_5_** | | | |  |
|  | b) Do you have enough energy for family and friends during leisure time? | | | | | | | | | | | **_1_** | | | | | | **_2_** | | | | | | **_3_** | | | | | **_4_** | | | | | | **_5_** | | | |  |
|  | c) How often are you emotionally exhausted? | | | | | | | | | | | **_1_** | | | | | | **_2_** | | | | | | **_3_** | | | | | **_4_** | | | | | | **_5_** | | | |  |
|  | d) Do you feel worn out at the end of the working day? | | | | | | | | | | | **_1_** | | | | | | **_2_** | | | | | | **_3_** | | | | | **_4_** | | | | | | **_5_** | | | |  |
|  | e) How often do you think: “I can’t take it anymore”? | | | | | | | | | | | **_1_** | | | | | | **_2_** | | | | | | **_3_** | | | | | **_4_** | | | | | | **_5_** | | | |  |
|  | f) How often do you feel weak and susceptible to illness? | | | | | | | | | | | **_1_** | | | | | | **_2_** | | | | | | **_3_** | | | | | **_4_** | | | | | | **_5_** | | | |  |
|  | g) How often are you physically exhausted? | | | | | | | | | | | **_1_** | | | | | | **_2_** | | | | | | **_3_** | | | | | **_4_** | | | | | | **_5_** | | | |  |
|  | h) How often do you feel tired? | | | | | | | | | | | **_1_** | | | | | | **_2_** | | | | | | **_3_** | | | | | **_4_** | | | | | | **_5_** | | | |  |
|  | i) Do you feel that every working hour is tiring for you? | | | | | | | | | | | **_1_** | | | | | | **_2_** | | | | | | **_3_** | | | | | **_4_** | | | | | | **_5_** | | | |  |
|  | j) How often do you feel worn out? | | | | | | | | | | | **_1_** | | | | | | **_2_** | | | | | | **_3_** | | | | | **_4_** | | | | | | **_5_** | | | |  |
|  |  | | | | | | | | | | | | | | | | | | | | | | | | | | | | | | | | | | | | | |  |
|  |  | | | | | | | | | **To a very low degree** | | | | | | | **To a low degree** | | | | | | **Somewhat** | | | | | **To a high degree** | | | | | | **To a very high degree** | | | | |  |
|  | k) Do you feel that you give more than you get back when you work with women and their families? | | | | | | | | | **_1_** | | | | | | | **_2_** | | | | | | **_3_** | | | | | **_4_** | | | | | | **_5_** | | | | |  |
|  | l) Does your work frustrate you? | | | | | | | | | **_1_** | | | | | | | **_2_** | | | | | | **_3_** | | | | | **_4_** | | | | | | **_5_** | | | | |  |
|  | m) Do you find it frustrating to work with women and their families? | | | | | | | | | **_1_** | | | | | | | **_2_** | | | | | | **_3_** | | | | | **_4_** | | | | | | **_5_** | | | | |  |
|  | n) Is your work emotionally exhausting? | | | | | | | | | **_1_** | | | | | | | **_2_** | | | | | | **_3_** | | | | | **_4_** | | | | | | **_5_** | | | | |  |
|  | o) Are you tired of working with women and families? | | | | | | | | | **_1_** | | | | | | | **_2_** | | | | | | **_3_** | | | | | **_4_** | | | | | | **_5_** | | | | |  |
|  |  | | | | | | | | | **To a very low degree** | | | | | | | **To a low degree** | | | | | | **Somewhat** | | | | | **To a high degree** | | | | | | **To a very high degree** | | | | |  |
|  | p) Does it drain your energy to work with women and their families? | | | | | | | | | **_1_** | | | | | | | **_2_** | | | | | | **_3_** | | | | | **_4_** | | | | | | **_5_** | | | | |  |
|  | q) Do you find it hard to work with women and their families? | | | | | | | | | **_1_** | | | | | | | **_2_** | | | | | | **_3_** | | | | | **_4_** | | | | | | **_5_** | | | | |  |
|  | r) Do you feel burnt out because of your work? | | | | | | | | | **_1_** | | | | | | | **_2_** | | | | | | **_3_** | | | | | **_4_** | | | | | | **_5_** | | | | |  |
|  | s) Do you sometimes wonder how long you will be able to continue working with women and their families? | | | | | | | | | **_1_** | | | | | | | **_2_** | | | | | | **_3_** | | | | | **_4_** | | | | | | **_5_** | | | | |  |
| **3.27** | **Currently, in an average week at the [organisation] how often would you get your scheduled breaks on each shift/day of work?** | | | | | | | | | | | | | | | | | | | | | | | | | | | | | | | | | | | | | |  |
|  | **_1_** | | 100% of shifts | | | | | | | | | | | | | | | | | | | | | | | | | | | | | | | | | | | |  |
|  | **_2_** | | 75% of shifts | | | | | | | | | | | | | | | | | | | | | | | | | | | | | | | | | | | |  |
|  | **_3_** | | 50% of shifts | | | | | | | | | | | | | | | | | | | | | | | | | | | | | | | | | | | |  |
|  | **_4_** | | 25% of shifts | | | | | | | | | | | | | | | | | | | | | | | | | | | | | | | | | | | |  |
|  | **_5_** | | 10% of shifts | | | | | | | | | | | | | | | | | | | | | | | | | | | | | | | | | | | |  |
|  | **_6_** | | 0% of shifts | | | | | | | | | | | | | | | | | | | | | | | | | | | | | | | | | | | |  |
| **3.28** | **In the last 12 months have you been absent from work, other than for holidays, or rostered days off (e.g. sick leave)?** | | | | | | | | | | | | | | | | | | | | | | | | | | | | | | | | | | | | | |  |
|  | **_1_** | Yes | | | | | | | | | | | | | | | | | | | | | | | | | | | | | | | | | | | | |  |
|  | **_2_** | No *(go to question 3.32)* | | | | | | | | | | | | | | | | | | | | | | | | | | | | | | | | | | | | |  |
| **3.29** | **Please estimate how many days of unplanned leave you have had in the last 12 months** | | | | | | | | | | | | | | | | | | | | | | | | | | | | | | | | | | | | | |  |
|  | **_1_** | <3 days | | | | | | | | | | | | | | | | | | | | | | | | | | | | | | | | | | | | |  |
|  | **_2_** | 3-5 days | | | | | | | | | | | | | | | | | | | | | | | | | | | | | | | | | | | | |  |
|  | **_3_** | 6-7 days | | | | | | | | | | | | | | | | | | | | | | | | | | | | | | | | | | | | |  |
|  | **_4_** | 1-2 weeks | | | | | | | | | | | | | | | | | | | | | | | | | | | | | | | | | | | | |  |
|  | **_5_** | Several weeks – indicate how many | | | | | | | | | | | | | | | | | | | | | | | | | | | | | | | | | | | | |  |
|  | **_6_** | Months – indicate how many | | | | | | | | | | | | | | | | | | | | | | | | | | | | | | | | | | | | |  |
| **3.30** | **What are the reasons for you taking unplanned leave? (tick all that apply)** | | | | | | | | | | | | | | | | | | | | | | | | | | | | | | | | | | | | | |  |
|  | **_1_** | | | Ill health (e.g. gastro, colds, infections) | | | | | | | | | | | | | | | | | | | | | | | | | | | | | | | | | | |  |
|  | **_2_** | | | Injuries unrelated to work (e.g. injury occurred while at home) | | | | | | | | | | | | | | | | | | | | | | | | | | | | | | | | | | |  |
|  | **_3_** | | | Work related injuries (e.g. injured back from work) | | | | | | | | | | | | | | | | | | | | | | | | | | | | | | | | | | |  |
|  | **_4_** | | | As a result of working a double shift within the last week | | | | | | | | | | | | | | | | | | | | | | | | | | | | | | | | | | |  |
|  | **_5_** | | | Personal-related stress | | | | | | | | | | | | | | | | | | | | | | | | | | | | | | | | | | |  |
|  | **_6_** | | | Mental health issues | | | | | | | | | | | | | | | | | | | | | | | | | | | | | | | | | | |  |
|  | **_7_** | | | Did not get roster request | | | | | | | | | | | | | | | | | | | | | | | | | | | | | | | | | | |  |
|  | **_8_** | | | Unable to swap shift when I needed to | | | | | | | | | | | | | | | | | | | | | | | | | | | | | | | | | | |  |
|  | **_9_** | | | Work-related fatigue | | | | | | | | | | | | | | | | | | | | | | | | | | | | | | | | | | |  |
|  | **_10_** | | | Feeling burnt out | | | | | | | | | | | | | | | | | | | | | | | | | | | | | | | | | | |  |
|  | **_11_** | | | Work-related stress | | | | | | | | | | | | | | | | | | | | | | | | | | | | | | | | | | |  |
|  | **_12_** | | | Have accepted a shift elsewhere when already rostered to work at the [organisation] | | | | | | | | | | | | | | | | | | | | | | | | | | | | | | | | | | |  |
|  | **_13_** | | | Caring for family member or friend | | | | | | | | | | | | | | | | | | | | | | | | | | | | | | | | | | |  |
|  | **_14_** | | | Work-related bullying | | | | | | | | | | | | | | | | | | | | | | | | | | | | | | | | | | |  |
|  | **_15_** | | | Childcare issues | | | | | | | | | | | | | | | | | | | | | | | | | | | | | | | | | | |  |
|  | **_16_** | | | Did not want to work a night duty shift | | | | | | | | | | | | | | | | | | | | | | | | | | | | | | | | | | |  |
|  | **_17_** | | | Was unable to get requested set shifts (e.g. can only work Monday, Tuesdays and Fridays) so took unplanned leave | | | | | | | | | | | | | | | | | | | | | | | | | | | | | | | | | | |  |
|  | **_18_** | | | Other - please describe: | | | | | | | | | | | | | | | | | | | | | | | | | | | | | | | | | | |  |
| **3.31** | **Of these ticked from question 3.30, which is the number one reason for you taking unplanned leave? (please choose one from question 3.30 and write down the number)** | | | | | | | | | | | | | | | | | | | | | | | | | | | | | | | | | | | | | |  |
|  |  | | | | | | | | | | | | | | | | | | | | | | | | | | | | | | | | | | | | | |  |
| **3.32** | **The following statements relate to your feelings in your current role.**  **Please check the most appropriate answer to each question. Where a statement does not apply to your current role please check ‘Not Applicable’.** | | | | | | | | | | | | | | | | | | | | | | | | | | | | | | | | | | | | | | |
|  |  | | | | | | | | | | **Strongly**  **Disagree** | | | | | **Disagree** | | | | | **Not Sure** | | | | | | **Agree** | | | | | | **Strongly Agree** | | | | | **N/A** | |
|  | a) I am confident that I have the skills for my current role | | | | | | | | | | **_1_** | | | | | **_2_** | | | | | **_3_** | | | | | | **_4_** | | | | | | **_5_** | | | | | **_99_** | |
|  | b) I have enough time to give women/families the care they need | | | | | | | | | | **_1_** | | | | | **_2_** | | | | | **_3_** | | | | | | **_4_** | | | | | | **_5_** | | | | | **_99_** | |
|  |  | | | | | | | | | | **Strongly**  **Disagree** | | | | | **Disagree** | | | | | **Not Sure** | | | | | | **Agree** | | | | | | **Strongly Agree** | | | | | **N/A** | |
|  | c) I need greater scope to provide women/ families with information about their care | | | | | | | | | | **_1_** | | | | | **_2_** | | | | | **_3_** | | | | | | **_4_** | | | | | | **_5_** | | | | | **_99_** | |
|  | d) There is not enough time to do my job properly | | | | | | | | | | **_1_** | | | | | **_2_** | | | | | **_3_** | | | | | | **_4_** | | | | | | **_5_** | | | | | **_99_** | |
|  | e) My current role allows me to plan care with women/families | | | | | | | | | | **_1_** | | | | | **_2_** | | | | | **_3_** | | | | | | **_4_** | | | | | | **_5_** | | | | | **_99_** | |
|  | f) Generally speaking, I am satisfied with my current role | | | | | | | | | | **_1_** | | | | | **_2_** | | | | | **_3_** | | | | | | **_4_** | | | | | | **_5_** | | | | | **_99_** | |
|  | g) I have plenty of opportunities to develop my skills as a nurse/midwife | | | | | | | | | | **_1_** | | | | | **_2_** | | | | | **_3_** | | | | | | **_4_** | | | | | | **_5_** | | | | | **_99_** | |
|  | h) I feel frustrated with my current role | | | | | | | | | | **_1_** | | | | | **_2_** | | | | | **_3_** | | | | | | **_4_** | | | | | | **_5_** | | | | | **_99_** | |
|  | i) I have plenty of opportunities to further my professional education | | | | | | | | | | **_1_** | | | | | **_2_** | | | | | **_3_** | | | | | | **_4_** | | | | | | **_5_** | | | | | **_99_** | |
|  | j) My current role allows me to provide women/families with choice about their care | | | | | | | | | | **_1_** | | | | | **_2_** | | | | | **_3_** | | | | | | **_4_** | | | | | | **_5_** | | | | | **_99_** | |
|  | k) I feel I am in a rut | | | | | | | | | | **_1_** | | | | | **_2_** | | | | | **_3_** | | | | | | **_4_** | | | | | | **_5_** | | | | | **_99_** | |
|  | l) I have limited opportunities to provide continuity of care | | | | | | | | | | **_1_** | | | | | **_2_** | | | | | **_3_** | | | | | | **_4_** | | | | | | **_5_** | | | | | **_99_** | |
|  | m) I lack professional support from my mangers | | | | | | | | | | **_1_** | | | | | **_2_** | | | | | **_3_** | | | | | | **_4_** | | | | | | **_5_** | | | | | **_99_** | |
|  | n) I have limited opportunities to provide women/families with individualised care | | | | | | | | | | **_1_** | | | | | **_2_** | | | | | **_3_** | | | | | | **_4_** | | | | | | **_5_** | | | | | **_99_** | |
|  | o) I get enough support from other clinical colleagues | | | | | | | | | | **_1_** | | | | | **_2_** | | | | | **_3_** | | | | | | **_4_** | | | | | | **_5_** | | | | | **_99_** | |
|  | p)I have enough professional independence | | | | | | | | | | **_1_** | | | | | **_2_** | | | | | **_3_** | | | | | | **_4_** | | | | | | **_5_** | | | | | **_99_** | |
|  | q) I have few opportunities to develop my skills as a nurse/midwife | | | | | | | | | | **_1_** | | | | | **_2_** | | | | | **_3_** | | | | | | **_4_** | | | | | | **_5_** | | | | | **_99_** | |
|  | r) My current role is very stressful | | | | | | | | | | **_1_** | | | | | **_2_** | | | | | **_3_** | | | | | | **_4_** | | | | | | **_5_** | | | | | **_99_** | |
|  | s) I have enough opportunities to make decisions about care | | | | | | | | | | **_1_** | | | | | **_2_** | | | | | **_3_** | | | | | | **_4_** | | | | | | **_5_** | | | | | **_99_** | |
|  | t) I have limited opportunities for professional development | | | | | | | | | | **_1_** | | | | | **_2_** | | | | | **_3_** | | | | | | **_4_** | | | | | | **_5_** | | | | | **_99_** | |
| **3.33** | **Does the [organisation] provide you with opportunities to consistently create an exceptional patient experience?** | | | | | | | | | | | | | | | | | | | | | | | | | | | | | | | | | | | | | |  |
|  | Never | | | | | | | Rarely | | | | | | Sometimes | | | | | | | | Frequently | | | | | | | | | Always | | | | | | | |  |
| **3.34** | **What would help you to be able to consistently create exceptional patient experiences?** | | | | | | | | | | | | | | | | | | | | | | | | | | | | | | | | | | | | | |  |
|  |  | | | | | | | | | | | | | | | | | | | | | | | | | | | | | | | | | | | | | |  |
| **3.35** | **What are the barriers that stop you from being able to consistently create exceptional patient experiences?** | | | | | | | | | | | | | | | | | | | | | | | | | | | | | | | | | | | | | |  |
|  |  | | | | | | | | | | | | | | | | | | | | | | | | | | | | | | | | | | | | | |  |
| **3.36** | **What do you feel the [organisation] needs to make it an exceptional place for you to work?** | | | | | | | | | | | | | | | | | | | | | | | | | | | | | | | | | | | | | |  |
|  |  | | | | | | | | | | | | | | | | | | | | | | | | | | | | | | | | | | | | | |  |
| **3.37** | **Do you feel that you are adequately acknowledged by this organisation for the work that you do?** | | | | | | | | | | | | | | | | | | | | | | | | | | | | | | | | | | | | | |  |
|  | **_1_** | | | | Yes | | | | | | | | | | | | | | | | | | | | | | | | | | | | | | | | | |  |
|  | **_2_** | | | | No | | | | | | | | | | | | | | | | | | | | | | | | | | | | | | | | | |  |
| **3.38** | **How would you like to be acknowledged by the [organisation]? (tick all that apply)** | | | | | | | | | | | | | | | | | | | | | | | | | | | | | | | | | | | | | |  |
|  | **_1_** | | | | I don’t want to be acknowledged by the [organisation] | | | | | | | | | | | | | | | | | | | | | | | | | | | | | | | | | |  |
|  | **_2_** | | | | Thank you email from management | | | | | | | | | | | | | | | | | | | | | | | | | | | | | | | | | |  |
|  | **_3_** | | | | Yearly awards for excellence | | | | | | | | | | | | | | | | | | | | | | | | | | | | | | | | | |  |
|  | **_4_** | | | | Food supplied by hospital | | | | | | | | | | | | | | | | | | | | | | | | | | | | | | | | | |  |
|  | **_5_** | | | | Personal acknowledgement from my manager | | | | | | | | | | | | | | | | | | | | | | | | | | | | | | | | | |  |
|  | **_6_** | | | | Indicated on social media | | | | | | | | | | | | | | | | | | | | | | | | | | | | | | | | | |  |
|  | **_7_** | | | | Monthly awards | | | | | | | | | | | | | | | | | | | | | | | | | | | | | | | | | |  |
|  | **_8_** | | | | Thank you cards | | | | | | | | | | | | | | | | | | | | | | | | | | | | | | | | | |  |
|  | **_9_** | | | | None of the above | | | | | | | | | | | | | | | | | | | | | | | | | | | | | | | | | |  |
|  | **_10_** | | | | Other - please describe: | | | | | | | | | | | | | | | | | | | | | | | | | | | | | | | | | |  |
| **3.39** | **From your experience at the [organisation] what is the “key message” that our Executive Director of nursing and midwifery needs to know?** | | | | | | | | | | | | | | | | | | | | | | | | | | | | | | | | | | | | | |  |
|  |  | | | | | | | | | | | | | | | | | | | | | | | | | | | | | | | | | | | | | |  |
| **3.40** | **Do you have anything further that you would like to add?** | | | | | | | | | | | | | | | | | | | | | | | | | | | | | | | | | | | | | |  |
|  |  | | | | | | | | | | | | | | | | | | | | | | | | | | | | | | | | | | | | | |  |
